# Role of Mitochondrial Retrograde Pathway in Regulating Ethanol-Inducible Filamentous Growth in Yeast

**DOI:** 10.3389/fphys.2017.00148

**Published:** 2017-03-29

**Authors:** Beatriz González, Albert Mas, Gemma Beltran, Paul J. Cullen, María Jesús Torija

**Affiliations:** ^1^Departament de Bioquímica i Biotecnologia, Universitat Rovira i VirgiliTarragona, Spain; ^2^Department of Biological Sciences, University at BuffaloBuffalo, NY, USA

**Keywords:** filamentous growth, pseudohyphal growth, quorum sensing, mitochondria-to-nucleus pathway, krebs cycle

## Abstract

In yeast, ethanol is produced as a by-product of fermentation through glycolysis. Ethanol also stimulates a developmental foraging response called filamentous growth and is thought to act as a quorum-sensing molecule. Ethanol-inducible filamentous growth was examined in a small collection of wine/European strains, which validated ethanol as an inducer of filamentous growth. Wine strains also showed variability in their filamentation responses, which illustrates the striking phenotypic differences that can occur among individuals. Ethanol-inducible filamentous growth in Σ1278b strains was independent of several of the major filamentation regulatory pathways [including fMAPK, RAS-cAMP, Snf1, Rpd3(L), and Rim101] but required the mitochondrial retrograde (RTG) pathway, an inter-organellar signaling pathway that controls the nuclear response to defects in mitochondrial function. The RTG pathway regulated ethanol-dependent filamentous growth by maintaining flux through the TCA cycle. The ethanol-dependent invasive growth response required the polarisome and transcriptional induction of the cell adhesion molecule Flo11p. Our results validate established stimuli that trigger filamentous growth and show how stimuli can trigger highly specific responses among individuals. Our results also connect an inter-organellar pathway to a quorum sensing response in fungi.

## Introduction

Fungal species represent a diverse group of microorganisms. Most fungal species exist in the wild. Other species live in commensal or pathogenic relationships with host organisms, while others still have been domesticated for food and technological benefits. *Saccharomyces sensu stricto* represents a group of highly related yeasts (Borneman and Pretorius, 2015). *Saccharomyces cerevisiae* and its relatives are commonly used in research laboratories and a variety of industrial processes. The ability of *Saccharomyces* to produce ethanol from several sugar sources makes it an essential component of the brewing and wine-making industries. Yeast not only produces ethanol as the major by-product of the alcoholic fermentation of sugars but also produces minor compounds such as aromatic (or fusel) alcohols that impart flavor and bouquet to wines. These properties have been studied to improve ethanol production and to understand the molecular basis of nutrient sensing and regulatory mechanisms in eukaryotes (Fleet and Heard, 1993; Ribéreau-Gayon et al., 2000; Beltran et al., 2004, 2008; Alper et al., 2006; Zaman et al., 2008).

Many fungal species, including yeasts, can undergo filamentous growth. Filamentous growth in yeast is a developmental foraging response, where cells become elongated and grow in connected chains (Gimeno et al., 1992; Kron et al., 1994). In some settings, cells can penetrate surfaces, which is known as invasive growth (Roberts and Fink, 1994). Some fungal species grow as multinucleate hyphae. Other species, like *S. cerevisiae*, produce pseudohyphae where cells undergo cytokinesis at each cell division. Filamentous growth has been extensively studied in yeast and other species, particularly pathogens, which require filamentous growth for virulence (Madhani and Fink, 1998; Lengeler et al., 2000; Polvi et al., 2015). Such studies have led to insights into the triggers, signaling pathways and transcriptional targets that control developmental responses in fungi and other eukaryotes.

One inducer of filamentous growth is nitrogen limitation (Gimeno et al., 1992). Another is the limitation of fermentable sugars like glucose (Cullen and Sprague, 2000). The morphogenetic response to limiting glucose is mediated by several pathways, including a mitogen-activated protein kinase pathway called the filamentous growth (fMAPK) pathway (Saito, 2010; Karunanithi and Cullen, 2012; Adhikari and Cullen, 2014; Adhikari et al., 2015), the AMP-dependent kinase AMPK Snf1p (Celenza and Carlson, 1989; Woods et al., 1994; Lesage et al., 1996; Cullen and Sprague, 2000; McCartney and Schmidt, 2001; Kuchin et al., 2002), and the RAS-cAMP-protein kinase A (PKA) pathway (Toda et al., 1985; Gimeno et al., 1992; Mosch et al., 1996, 1999; Colombo et al., 1998; Robertson and Fink, 1998a,b; Rupp et al., 1999b; Robertson et al., 2000; Pan and Heitman, 2002). Filamentous growth is also regulated by the Rim101 pathway, which regulates the response to pH (Lamb et al., 2001; Lamb and Mitchell, 2003; Barrales et al., 2008). Other regulators include the chromatin remodeling complex Rpd3(L) (Carrozza et al., 2005; Barrales et al., 2008; Ryan et al., 2012), the tRNA modification complex Elongator (Krogan and Greenblatt, 2001; Winkler et al., 2001; Petrakis et al., 2004; Li et al., 2007; Svejstrup, 2007), and the Pho80p-Pho85p cyclin and cyclin-dependent kinase (Measday et al., 1997; Huang et al., 2002, 2007; Shemer et al., 2002; Moffat and Andrews, 2004; Chavel et al., 2014). In addition to these pathways, genetic (Lorenz and Heitman, 1998; Palecek et al., 2000), genomic and proteomic screens (Jin et al., 2008; Xu et al., 2010; Ryan et al., 2012) have identified many other proteins and pathways that impact filamentous growth. Thus, filamentous growth resembles cell differentiation in metazoans, where global reorganization of cellular processes results in the construction of a new cell type.

Fungal species also utilize small molecules to interpret information about their environment. Like many other microbial species (Miller and Bassler, 2001; Parsek and Greenberg, 2005; Rumbaugh et al., 2009), *S. cerevisiae* exhibits quorum-sensing responses (Hlavacek et al., 2009; Prunuske et al., 2012). Yeast can sense and respond to ammonia (Palkova et al., 1997), aromatic (fusel) alcohols (Chen and Fink, 2006), and ethanol (Dickinson, 1994, 1996; Lorenz et al., 2000). By products of the Ehrlich reactions (Hazelwood et al., 2008), fusel alcohols are formed by conversion of several amino acids into glutamate as a nitrogen source under nitrogen-limiting conditions (Ljungdahl and Daignan-Fornier, 2012). Fusel alcohols are produced at higher levels in nitrogen-limiting medium and sensed in a density-dependent manner by a PKA-dependent mechanism to regulate filamentous growth (Chen and Fink, 2006). Multiple fungal species produce and sense a variety of aromatic alcohols, which may impart selectivity in this type of cellular communication (Chen et al., 2004; Chen and Fink, 2006; Sprague and Winans, 2006; Kruppa, 2008; Langford et al., 2013). Recent efforts have expanded the diversity alcohols that can be sensed and measured their impact on fungal behavioral responses (Ghosh et al., 2008; Wuster and Babu, 2010; Sharma and Prasad, 2011; Albuquerque and Casadevall, 2012; Bojsen et al., 2012; Avbelj et al., 2015; Williams et al., 2015). An open question has been to identify the regulatory pathways that control alcohol-mediated morphogenesis and understand how cells detect and respond to these stimuli. Addressing this problem has a practical benefit, as industrial manipulation of yeast may be accelerated by understanding density-dependent growth and behavioral responses (Westman and Franzen, 2015).

To better understand common and unique elements of the filamentous growth response, a diverse collection of strains was examined from the “wine/European” group (Goffeau et al., 1996; Wei et al., 2007; Borneman et al., 2008, 2011; Argueso et al., 2009; Liti et al., 2009; Novo et al., 2009). Most strains tested underwent filamentous growth in response to limiting glucose, limiting nitrogen, or the presence of ethanol or fusel alcohols. A specific role for the mitochondrial retrograde (RTG) pathway, which controls the response to compromised mitochondrial function (Liu and Butow, 2006) and is known to regulate filamentous growth (Jin et al., 2008; Chavel et al., 2010, 2014; Aun et al., 2013; Starovoytova et al., 2013), was identified as a specific regulator of ethanol-inducible invasive growth. RTG regulated TCA cycle flux in response to ethanol to modulate filamentous growth. Thus, the study connects an inter-organellar signaling pathway to a quorum-sensing morphogenetic response in fungi.

## Materials and methods

### Yeast strains, media, and growth conditions

Yeast strains are described in Table 1. Standard media was used (Rose et al., 1990). Yeast strains were generated by polymerase chain reaction (PCR)-based homologous recombination techniques using auxotrophic or antibiotic resistant markers (Goldstein and McCusker, 1999). Yeast were grown on YPD (2% peptone, 1% yeast extract, 2% glucose, and 2% agar), minimal medium [(MM) 1X Yeast Nitrogen Base (YNB) without amino acids or ammonium, 2% glucose, and 10 mM (NH_4_)_2_SO_4_], synthetic media [(SD) 1X YNB, 2% glucose, and 37 mM (NH_4_)_2_SO_4_] with ammonium and dextrose [(SAD) 1X YNB, 1% glucose, and 37 mM (NH_4_)_2_SO_4_], or with ammonium and low glucose [(SALG) 1X YNB, 0.5% glucose, and 37 mM (NH_4_)_2_SO_4_]. To evaluate pseudohyphal growth, yeast were grown on synthetic medium with dextrose and low-ammonium [(SLAD) 1X YNB, 2% glucose, 50 μM (NH_4_)_2_SO_4_, and 2% agar (Gimeno et al., 1992)]. Media was supplemented with uracil for auxotrophic mutants. For some experiments, SD and SLAD media were supplemented with 500 μM tryptophol, tyrosol, or phenylethanol and 2%(v/v) ethanol. The *CIT2-lacZ* plasmid has been described (Liu and Butow, 1999) and was provided by Dr. Zhengchang Liu (Louisiana State University, New Orleans). Beta-galactosidase assays were performed as described (Chavel et al., 2014).

**Table 1:** **Yeast strains used in the study**.

**Strain**	**Genotype**	**References**
S288c	*MAT*α *SUC2 gal2 mal2 mel flo1 flo8-1 hap1 ho bio1 bio6*	Mortimer and Johnston, 1986
Nsa^a^	*MAT***a***/MATα*	Wang et al., 2015
S1^b^	*MAT***a***/MATα*	Padilla et al., 2016
QA23^c^	*MAT***a***/MATα*	Borneman et al., 2011
T73^d^	*MAT***a***/MATα*	Querol et al., 1992
SB	*HO/HO, asp1-H142/asp1-H142*	Marullo et al., 2007
P5^e^	*MAT***a***/MATα*	García-Ríos et al., 2014
P24^c^	*MAT***a***/MATα*	García-Ríos et al., 2014
VIN7^f^	*Triploid allohybrid S. cerevisiae × S. kudriavzevii*	Borneman et al., 2012
W27^c^	*Hybrid S.cerevisiae × S. kudriavzevii*	Schütz and Gafner, 1994
PC312^g^	*MATα ura3-52*	Liu et al., 1993
PC313	*MAT***a** *ura3-52*	Liu et al., 1993
PC318	*MATα ura3-52 rho0*	Chavel et al., 2010
PC344	*MAT***a***/MATα ura3-52/ura3-52*	Cullen and Sprague, 2000
PC443	*MAT***a** *ste4 FUS1-lacZ FUS1-HIS3 ura3-52 snf1::URA3*	Cullen and Sprague, 2000
PC471	*MAT***a** *ste4 FUS1-lacZ FUS1-HIS3 ura3-52 bud6::KlURA3*^h^	Cullen and Sprague, 2002
PC538	*MAT***a** *ste4 FUS1-lacZ FUS1-HIS3 ura3-52*	Cullen et al., 2004
PC539	*MAT***a** *ste4 FUS1-lacZ FUS1-HIS3 ura3-52 ste12::KLURA3*	Cullen et al., 2004
PC549	*MAT***a** *ste4 FUS1-lacZ FUS1-HIS3 ura3-52 ste20::URA3*	Cullen and Sprague, 2000
PC563	*MAT***a** *ste4 FUS1-lacZ FUS1-HIS3 ura3-52 bud8::KlURA3*	Cullen and Sprague, 2002
PC611	*MAT***a** *ste4 FUS1-lacZ FUS1-HIS3 ura3-52 ste11::URA3*	Cullen et al., 2004
PC999	*MAT***a** *ste4 FUS1-lacZ FUS1-HIS3 ura3-52 MSB2-HA*	Cullen et al., 2004
PC2549	*MAT***a** *ura3-52 ras2::KlURA3*	Chavel et al., 2010
PC2584	*MATa ste4 FUS1-lacZ FUS1-HIS3 ura3-52 tpk1::NAT*	Chavel et al., 2010
PC2763	*MAT***a** *ste4 FUS1-lacZ FUS1-HIS3 ura3-52 elp2::KlURA3*	Abdullah and Cullen, 2009
PC2953	*MAT***a** *ste4 FUS1-lacZ FUS1-HIS3 ura3-52 MSB2-HA rim101::ura3*	Chavel et al., 2010
PC3030	*MAT***a** *ste4 FUS1-lacZ FUS1-HIS3 ura3-52 MSB2-HA sin3::NAT*	Chavel et al., 2010
PC3035	*MAT***a** *ste4 FUS1-lacZ FUS1-HIS3 ura3-52 MSB2-HA mks1::NAT*	Chavel et al., 2010
PC3097	*MAT*α *ura3-52 leu2 pex3::HYG*	This study
PC3363	*MAT***a** *ste4 FUS1-lacZ FUS1-HIS3 ura3-52 MSB2-HA nrg1::KLURA3*	Chavel et al., 2010
PC3642	*MAT***a** *ste4 FUS1-lacZ FUS1-HIS3 ura3-52 MSB2-HA rtg3::NAT*	Chavel et al., 2010
PC3643	*MAT***a** *ste4 FUS1-lacZ FUS1-HIS3 ura3-52 MSB2-HA tco89::NAT*	Chavel et al., 2014
PC3652	*MAT***a** *ste4 FUS1-lacZ FUS1-HIS3 ura3-52 MSB2-HA rtg2::NAT*	Chavel et al., 2010
PC3654	*MAT***a** *ste4 FUS1-lacZ FUS1-HIS3 ura3-52 MSB2-HA tor1::NAT*	Chavel et al., 2010
PC3695	*MAT***a** *ste4 FUS1-lacZ FUS1-HIS3 ura3-52 MSB2-HA rtg1::NAT*	Chavel et al., 2014
PC3909	*MAT***a** *ste4 FUS1-lacZ FUS1-HIS3 ura3-52 ste12::KLURA3 mks1::NAT*	This study
PC3910	*MAT***a** *ste4 FUS1-lacZ FUS1-HIS3 ura3-52 ste20::URA3 mks1::NAT*	This study
PC3911	*MAT***a** *ste4 FUS1-lacZ FUS1-HIS3 ura3-52 ste11::URA3 mks1::NAT*	This study
PC4041	*MAT***a** *ste4 FUS1-lacZ FUS1-HIS3 ura3-52 MSB2-HA rtg2::NAT ssk1::KlURA3*	This study
PC4141	*MATa ste4 FUS1-lacZ FUS1-HIS3 ura3-5 tpk2::URA3*	Chavel et al., 2014
PC5059	*MAT***a** *ste4 FUS1-lacZ FUS1-HIS3 ura3-52 mig2::HYG*	This study
PC5084	*MATa ste4 FUS1-lacZ FUS1-HIS3 ura3-52 Msb2-HA tpk3::NAT*	Chavel et al., 2014
PC5582	*MAT***a** *ste4 FUS1-lacZ FUS1-HIS3 ura3-52 pbs2::KanMX6*	This study
PC5594	*MAT***a** *ste4 FUS1-lacZ FUS1-HIS3 ura3-52 flo11::KlURA3*	This study
PC5864	*MAT***a** *ste4 FUS1-lacZ FUS1-HIS3 ura3-52 sch9::KlURA3*	This study
PC6017^i^	*MAT*α *can1Δ::Ste2pr-spHIS5 lyp1Δ::Ste3pr-LEU2 his3::hisG leu2Δ0 ura3Δ0*	Ryan et al., 2012
PC6018	*MAT***a***/MATα can1Δ::Ste2pr-spHIS5/can1Δ::Ste2pr-spHIS5 lyp1Δ::Ste3pr-LEU2/lyp1Δ::Ste3pr-LEU2 his3::hisG/his3::hisG leu2Δ0/leu2Δ0 ura3Δ0/ura3Δ0*	Ryan et al., 2012

a *Natural isolate from wine*.

b *Natural isolate from wine (CECT 13132)*.

c *Commercial wine yeast Lalvin® Lallemand*.

d *Commercial wine yeast Lalvin® Lallemand (CECT1894)*.

e *Commercial wine yeast Lalvin® ICVGRE Lallemand*.

f *Commercial wine yeast AWRI1539®*.

g *All PC strains are in the Σ1278b strain background*.

h *KlURA3 refers to the Kluyveromyces lactis URA3 gene cassette*.

i *Mutants derived from this strain were constructed in a genomic collection and were also tested in the study*.

### Pseudohyphal growth assays

Examination of pseudohyphae was determined as described (Gimeno et al., 1992). Strains were grown for 16 h at 28°C in MM and harvested by centrifugation (1,000 rpm for 3 min). To obtain single colonies, cells were diluted by a factor of 10^6^ in sterile water, and 100 μL of cells were spread onto media (SAD, SALG, and SLAD). Plates were incubated at 28°C and observed daily for 10 d by microscopy for colony morphology.

### Invasive growth assays

Strains were grown for 16 h at 30°C in MM, harvested by centrifugation (10,000 rpm for 3 min) at an optical density (O.D. A_600_) of 2.0, washed once in sterile water and resuspended in sterile water. Ten microliters of cells were spotted on semisolid agar media. Plates were incubated at 28°C. Invasive growth was determined by the plate-washing assay (Roberts and Fink, 1994). Colonies were photographed before and after washing over a 10 days period. Plates were washed in a stream of water (soft wash) and colonies were rubbed from the surface with a gloved finger (hard wash). ImageJ (http://rsb.info.nih.gov/ij/) was used to quantitate invasive growth (Zupan and Raspor, 2008). Background intensity was determined for each spot and subtracted from the densitometry of the area of invaded cells. Densitometric analysis was performed on invasive patches over multiple days. Tukey's *t*-test was used to determine statistical significance and generate *p*-values. The Shapiro-Wilk and Jarque-Bera normality tests showed that the data fit a normal distribution. A non-parametric statistics test (Wilcoxon test) showed the same results as the Tukey's *t*-test.

### Quantitative polymerase chain reaction (qPCR) analysis

Quantitative PCR was performed as described (Beltran et al., 2004). Ethanol addition stimulated the expression of *FLO11* at all-time points except 24 h. Strains were grown in MM for 24 h at 28°C, washed with MiliQ sterile water (Millipore Q-PODTM Advantage A10) and resuspended in the indicated media at an O.D. A_600_ of 2.0. Cells were inoculated in SLAD media and in SAD media, and samples were taken at 2 h. To study the effect of nitrogen concentration in *FLO11* expression, strains were grown in MM for 24 h at 28°C, washed with MiliQ sterile water (Millipore Q-PODTM Advantage A10) and resuspended in SAD and SLAD media at an O.D. A_600_ of 2.0. Samples were taken at 2 h to analyze the *FLO11* expression. To study the effect of ethanol in *FLO11* expression, cells were inoculated at an O.D. A_600_ of 2.0 in SLAD medium with or without ethanol (2% v/v) Samples were taken at 45 min, 2, 8, and 24 h. RNA extraction was performed using an RNeasy Mini Kit (Qiagen). RNA concentration was adjusted to 320 ng/μL. Reverse transcription was performed using SuperScript® III Reverse Transcriptase (Invitrogen) and Oligo (dt) 20 Primer (Invitrogen).

qPCR was performed using an Applied Biosystems 7300 Fast Real-Time PCR System (Applied Biosystems, USA). SyberGreen master mix was used according to the manufacturer's instructions (Applied Biosystems, USA). Reactions contained 25 μL sample (5 μL cDNA, 1 μM each primer, 10 μL SyberGreen master mix, H_2_0 q.s.p. 25 μL). The starting quantity of genes was normalized with *ACT1* (Chavel et al., 2010). Relative gene expression was calculated using the 2^−Δ**Ct**^ formula, where Ct is defined as the cycle at which fluorescence was determined to be statistically significant above background; ΔCt is the difference in Ct of the *FLO11* gene and housekeeping gene (*ACT1*). The primers used were *FLO11* forward (5′-CACTTTTGAAGTTTATGCCACACAAG-3′) and *FLO11* reverse (5′-CTTGCATATTGAGCGGCACTAC-3′) based on Chen and Fink (2006), and *ACT1* forward (5′-TGGATTCCGGTGATGGTGTT-3′) and *ACT1* reverse (5′-CGGCCAAATCGATTCTCAA-3′).

### Microscopy

Differential-interference-contrast (DIC) and bright-field microscopy was performed using an Axioplan 2 fluorescent microscope (Zeiss) with a PLAN-APOCHROMAT 100X/1.4 (oil) objective (N.A. 0.17). Digital images were obtained with the Axiocam MRm camera (Zeiss). Axiovision 4.4 software (Zeiss) was used for image acquisition and analysis and for rendering 3D Z-stack images. Images were further analyzed in Adobe Photoshop, where adjustments of brightness and contrast were made.

## Results

### Exploring filamentous growth in a collection of wild and industrial yeast strains

To understand the common and unique features of filamentous growth in yeast, a collection of wild and industrial yeast strains used in wine making was examined (Table 1). Strains were compared to ∑1278b, a well-characterized strain background that undergoes filamentous growth (Gimeno et al., 1992), and S288c, which is commonly used in research laboratories (Mortimer and Johnston, 1986) but has acquired mutations due to genetic manipulation that render it unable to undergo filamentous growth (Liu et al., 1996; Dowell et al., 2010; Chin et al., 2012).

**Figure 1:**
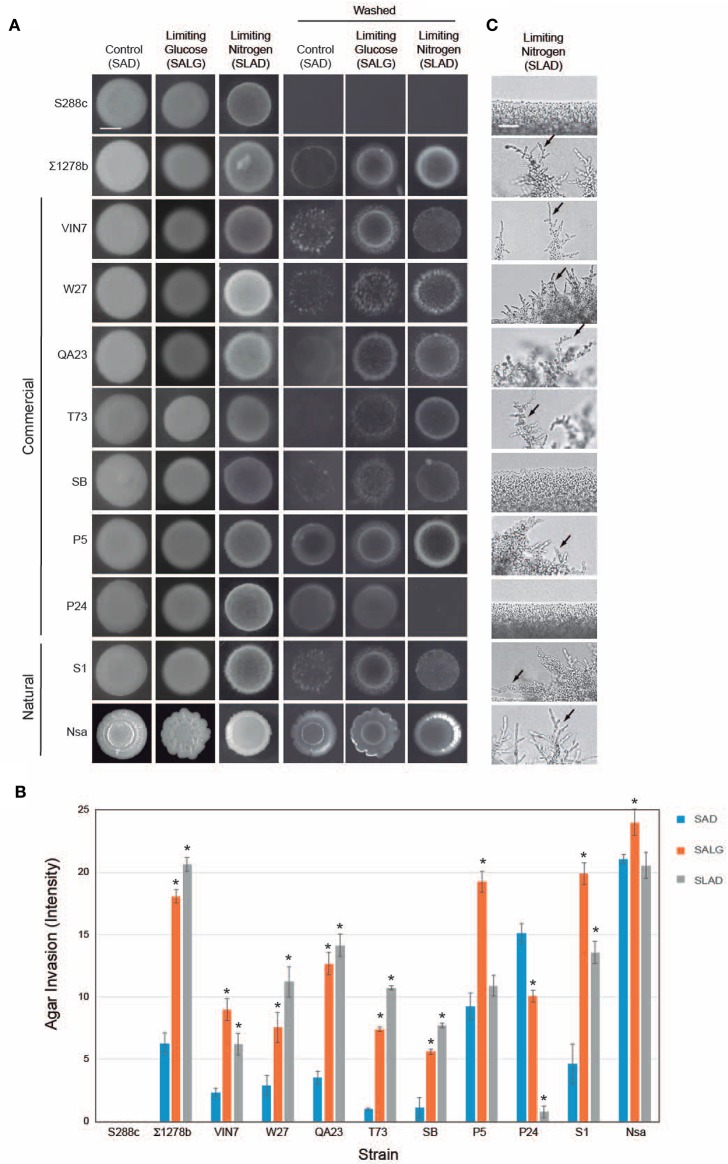
**Filamentous growth phenotypes of wine strains. (A)** Plate-washing assay (PWA). Equal concentrations of cells (OD_600 nm_ = 2) were spotted in 10 μL aliquots onto the indicated media. Plates were incubated for 5 days at 30°C and washed in a stream of water. Bar, 5 mm. **(B)** Quantitation of invasive growth in panel **(A)** by densitometry. Cells were spotted in triplicate, and the average values are shown. Error bars represent the standard difference between experiments. Asterisk denotes a *p* < 0.01 for samples relative to each strain's invasion in SAD. **(C)** Pseudohyphal growth of micro-colonies. Cells were grown for 3 days in minimal medium (MM) at 30°C, diluted by a factor of 10^6^ and spotted onto SLAD media. Plates were incubated for 5 days. Colonies were examined by microscopy at 40X magnification. A representative image is shown. Bar, 25 μm. Arrows mark examples of pseudohyphae.

One aspect of filamentous growth is invasive growth, which can be assessed by the plate-washing assay (PWA), and which measures penetration of filamentous cells into surfaces (Roberts and Fink, 1994). Invasive growth in nutrient-rich (SAG) conditions was compared to conditions that induce filamentous growth, nitrogen limitation (SLAD; Gimeno et al., 1992) and glucose limitation (SALG; Cullen and Sprague, 2000) as shown in Figure 1A. The results were quantitated by densitometric analysis (Figure 1B). As expected, S288c did not undergo invasive growth, and ∑1278b underwent invasive growth that was higher in media lacking glucose or nitrogen (Figure 1A, washed and Figure 1B). Most wine strains underwent invasive growth, which was stimulated in nitrogen- and glucose-limited medium (including VIN7, W27, QA23, T73, SB, and S1; Figures 1A,B). Three strains showed a different trend: P5 invaded equally well in glucose-rich and glucose-limiting media, P24 did not invade nitrogen-limiting medium, and Nsa showed constitutive invasion. Moreover, the pattern of invasive growth varied widely among strains (Figure 1A).

Another aspect of filamentous growth is pseudohyphal growth, which can be measured by microscopic examination of colony peripheries (Gimeno et al., 1992). As expected, S288c did not form pseudohyphae, and ∑1278b formed pseudohyphae in nitrogen-limiting medium (Figure 1C, SLAD). Most strains formed pseudohyhae in nitrogen-limiting media (Figure 1C, including VIN7, W27, QA23, T73, SB, P5, S1, and Nsa), except SB, which did not form pseudohyphae until day 16 (for Figure 1C, day 5 is shown) and P24, which did not form pseudohyphae by day 20 when the experiment was terminated. The pattern of pseudohyphae varied among strains. With the exception of Nsa, which formed pseudohyphae in glucose- (Figure S1A, Nsa SALG, arrow) and nitrogen-limiting media, all other strains formed pseudohyphae exclusively under nitrogen-limitng conditions. Invasive and pseudohyphal growth require cell adhesion mediated by the flocculin Flo11p (Lambrechts et al., 1996; Lo and Dranginis, 1996; Guo et al., 2000). *FLO11* expression is induced during filamentous growth (Rupp et al., 1999a). A subset of wine strains that were tested all showed induction of *FLO11* expression under nitrogen-limiting conditions (Figure S1B). Therefore, above results agree with the widely accepted notion that glucose and nitrogen limitation are general inducers of filamentous growth.

**Figure 2:**
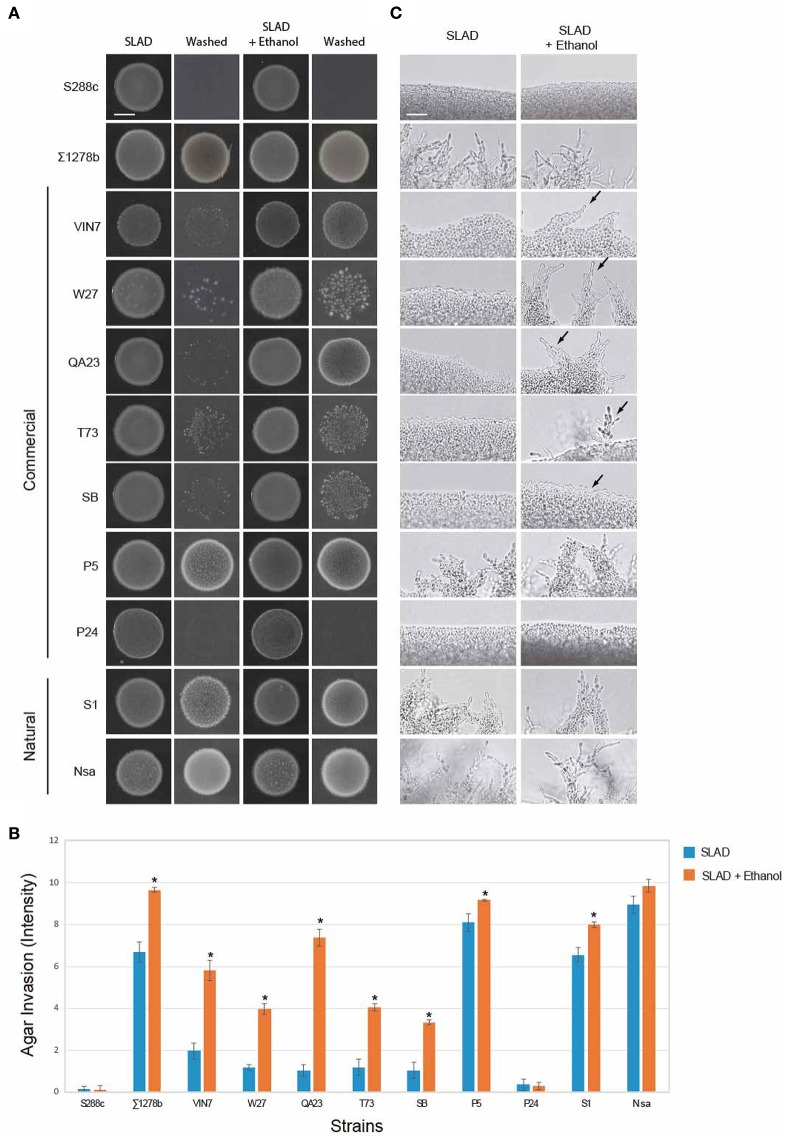
**Response of wine strains to ethanol. (A)** PWA of cells spotted onto nitrogen-limited medium (SLAD) with or without ethanol (2% v/v). Plates were incubated for 2 days at 30°C and washed in a stream of water. Bar, 5 mm. **(B)** Quantitation of invasive growth in panel **(A)** by densitometry, performed as described in Figure 1B. Cells were spotted in triplicate, and the average values are shown. Error bars represent the standard difference between experiments. Asterisk denotes a *p* < 0.01 for samples relative to each strain's invasion in SLAD. **(C)** Microscopy of colony perimeters with or without ethanol at 40X magnification. Bar, 25 μm. Arrows mark examples of pseudohyphae.

Ethanol also stimulates filamentous growth (Dickinson, 1994, 1996; Lorenz et al., 2000). Ethanol induced filamentous growth specifically in nitrogen-limiting medium (Figure S2A) and showed a maximal effect at a concentration of 2% (Figure S2B). At this concentration, ethanol did not impact growth (Figure S2C; yeast can survive in 12% ethanol; Lleixà et al., 2016). Thus, tests were performed at 2% ethanol in nitrogen-limiting media. As expected, S288c did not show invasive growth by the addition of ethanol (Figures 2A–C), and ∑1278b showed ethanol-inducible invasive growth (Figures 2A,B). In particular, cells invaded the agar more robustly (Figures 2A,B), and pseudohyphae formed at earlier time points (Figure 2C, colonies were grown for 2 days compared to 5 days in Figure 1C). With the exception of P24 and Nsa, most strains showed increased invasive growth in response to ethanol (Figures 2A–C including VIN7, W27, QA23, T73, SB, S1, and P5). By these criteria, ethanol can also be viewed as a general inducer of filamentous growth. The fusel alcohol tryptophol stimulates filamentous growth in ∑1278b strains (Figure S3; Chen and Fink, 2006). Tryptophol stimulated invasive growth of most wine strains in nitrogen-rich (SAD) but not nitrogen-limiting (SLAD) medium (Figure S3, including VIN7, W27, QA23, T73, and S1). Thus, in line with previous studies, fusel alcohols like tryptophol are general inducers of filamentous growth.

### Major filamentation regulatory pathways are not required for ethanol-inducible filamentous growth

**Figure 3:**
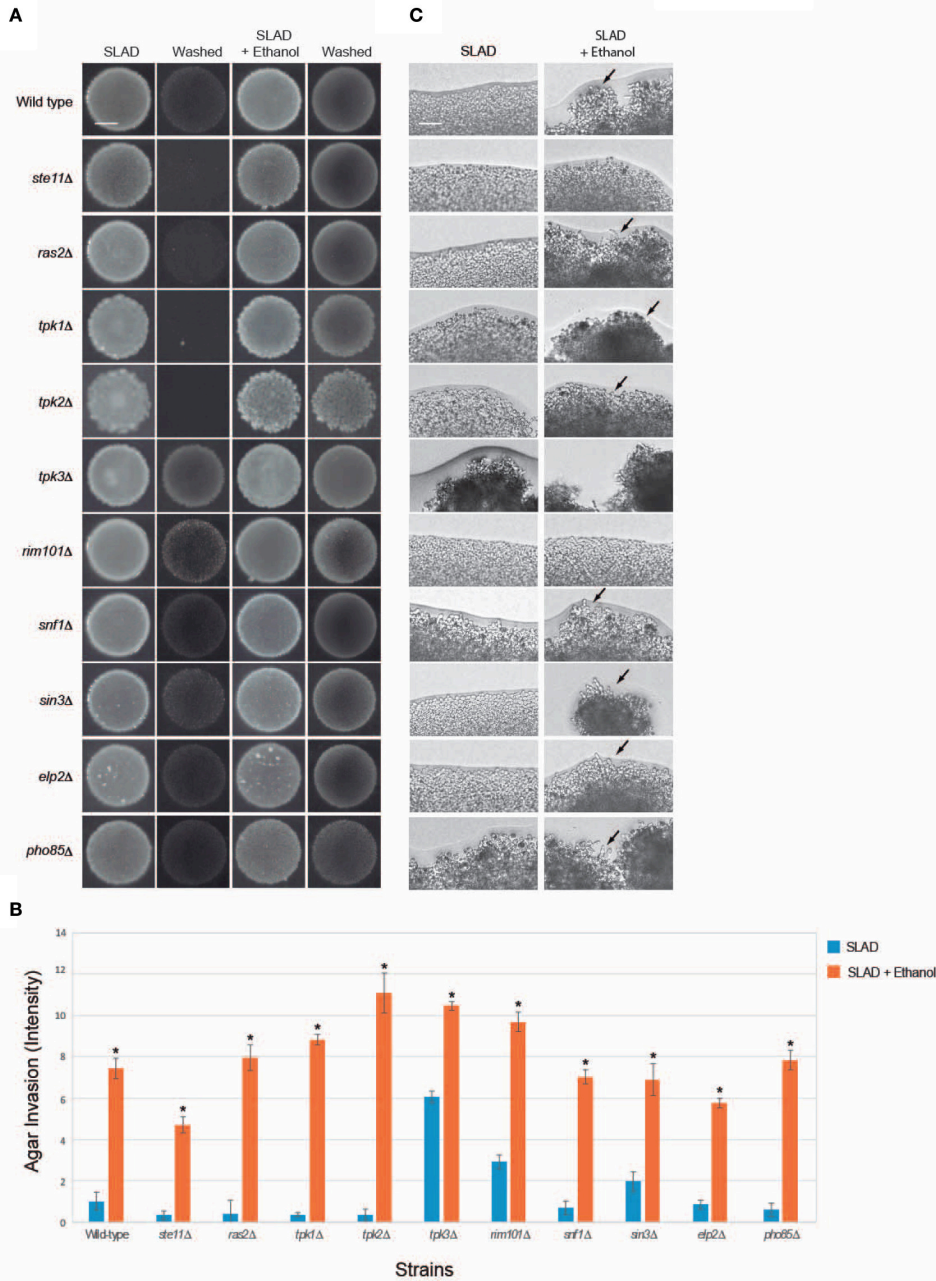
**Evaluating mutants lacking established filamentation regulatory pathways for ethanol-inducible invasion. (A)** Wild-type cells (PC538, Σ1278b *MAT***a** haploid) and the indicated isogenic mutants were spotted onto nitrogen-limited medium (SLAD) with or without 2% ethanol (v/v). Plates were incubated for 4 days at 30°C, photographed, washed in stream of water, and photographed again. Bar, 5 mm. **(B)** Quantitation of invasive growth in panel **(A)** by densitometry, performed as described in the legend for Figure 1B. Cells were spotted in triplicate, and the average values are shown. Error bars represent the standard difference between experiments. Asterisk denotes a *p* < 0.01 for samples relative to each strain's invasion in SLAD. **(C)** Colony peripheries from the plates in panel **(A)** were examined at 20X magnification. Bar, 50 μm. Arrows mark examples of pseudohyphae.

We focused on ethanol-inducible filamentous growth because ethanol was a stronger inducer of filamentous growth than fusel alcohols. How ethanol is sensed and triggers filamentous growth has not been extensively studied. The ethanol response occurred in diploid (Figure 2) and haploid (Figure 3) strains of the ∑1278b background, which facilitated genetic analysis of the response.

Signaling pathways known to regulate filamentous growth were tested for a role in regulating ethanol-inducible filamentous growth. Specifically, mutants were tested that lack key regulators of fMAPK (*ste11*Δ; Ste11p is the MAPKKK), Ras2p-cAMP-PKA (*ras2*Δ) and PKA (Tpk in yeast) subunits Tpk1p, Tpk2p, and Tpk3p (*tpk1*Δ, *tpk2*Δ, and *tpk3*Δ), Snf1p (*snf1*Δ), Rim101p (*rim101*Δ), Rpd3p(L) (*sin3*Δ), Elongator (*elp2*Δ), and Pho85p (*pho85*Δ). Surprisingly, all of the mutants showed enhanced invasive growth in media containing ethanol (Figures 3A,B). The examination of colony perimeters generally bore this out, either showing enhanced filament formation or clumpiness (Figure 3C, arrows), which is indicative of elevated cell-cell adhesion. Colony perimeters did not show a change for the *ste11*Δ and *rim101*Δ mutants. Thus, fMAPK and Rim101 pathways may play some role in mediating ethanol-dependent filamentous growth. In summary these results show that ethanol exerts its effect on filamentous growth independent of several of the major regulatory pathways that control filamentous growth.

Unexpectedly, several mutants did not show an invasive growth defect in SLAD media. Specifically, the *rim101*Δ, *sin3*Δ, *snf1*Δ, *elp2*Δ, and *pho85*Δ mutants invaded the agar as well as or better then wild-type cells [Figures 3A,B; *tpk3*Δ is not defective for invasive growth (Robertson and Fink, 1998a; Robertson et al., 2000; Chavel et al., 2010)]. We have previously shown that the *rim101*Δ (Chavel et al., 2014), *sin3*Δ (Chavel et al., 2010), *snf1*Δ (Cullen and Sprague, 2000), *elp2*Δ (Abdullah and Cullen, 2009), and *pho85*Δ (Chavel et al., 2014) mutants have an invasive growth defect on rich media, and we verified that phenotype here (Figure S4A; YPD). Thus, there may be differences in the roles these pathways play in regulating invasive growth depending on growth on YPD or SLAD. This hypothesis is consistent with the fact that mutants scored for pseudohyphal and invasive growth do not completely overlap in a genome-wide screen (Ryan et al., 2012) and with the fact that several pathways, like Snf1p, play different roles in response to carbon and nitrogen limitation (Orlova et al., 2010).

### Mitochondrial retrograde pathway is required for ethanol-inducible invasive growth

**Figure 4:**
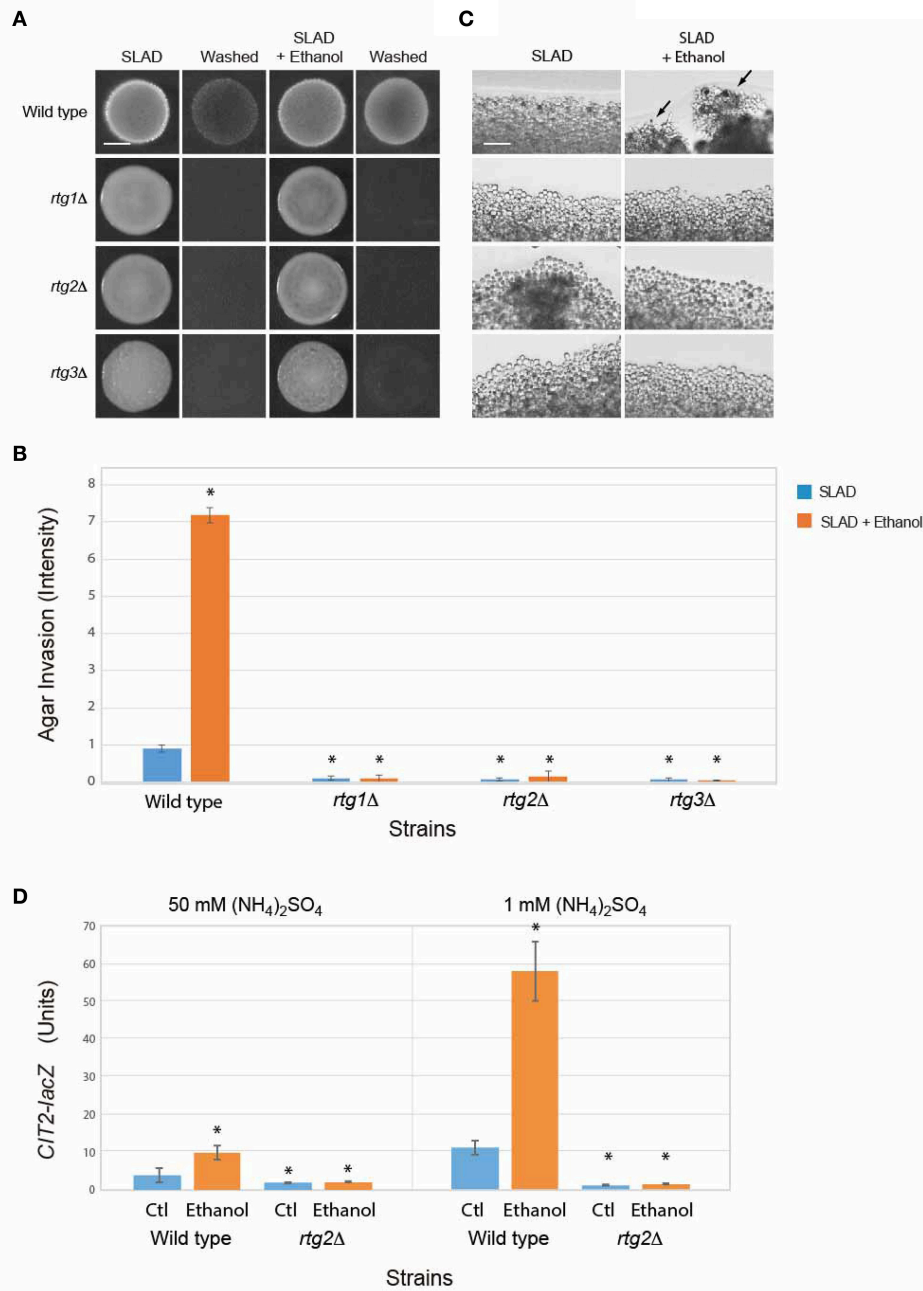
**Role of the RTG pathway in regulating ethanol-inducible invasive growth. (A)** Wild-type cells (PC538, Σ1278b *MAT***a** haploid) and the indicated isogenic mutants were spotted onto nitrogen limiting medium (SLAD) with or without 2% ethanol (v/v). Plates were incubated for 4 days at 30°C, photographed, washed in stream of water, and photographed again. Bar, 5 mm. **(B)** Quantitation of invasive growth in panel **(A)** by densitometry, performed as described in the legend for Figure 1B. Cells were spotted in triplicate, and the average values are shown. Error bars represent the standard difference between experiments. Asterisk denotes a *p* < 0.01 for samples relative to wild type in SLAD. **(C)** Colony peripheries from the plates in panel **(A)** were examined by microscopy at 20X magnification. Bar, 50 μm. Arrows mark examples of pseudohyphae. **(D)** Beta-galactosidase activity of the *CIT2-lacZ* reporter in wild-type cells and the *rtg2*Δ mutant grown in 1 or 50 mM (NH_4_)_2_SO_4_ with or without 2% ethanol (v/v). Experiments were performed in triplicate from independent inductions. Error bars represent the standard deviation between experiments. Asterisk denotes a *p* < 0.01 for samples relative to wild type in media lacking ethanol (Ctl).

Other proteins and pathways regulate filamentous growth than those tested above (Ryan et al., 2012). A broader collection of genes implicated in filamentous growth regulation was examined. One of these is the mitochondrial retrograde pathway (or RTG pathway; Sekito et al., 2002; Liu et al., 2003; Liu and Butow, 2006; Kleine and Leister, 2016), which senses changes in metabolic respiration (Aun et al., 2013) to regulate filamentous growth. The RTG pathway has recently been shown to regulate the filamentation response to the alcohol butanol (Starovoytova et al., 2013). Rtg2p is a positive regulator of the retrograde pathway (Ferreira Junior et al., 2005). The *rtg2*Δ mutant was defective for ethanol-dependent invasive growth (Figures 4A–C). The RTG pathway is composed of two other regulators, the basic helix-loop-helix leucine zipper transcription factors Rtg1p and Rtg3p, which hetero-dimerize to regulate transcription (Jia et al., 1997). The *rtg1*Δ and *rtg3*Δ mutants were also defective for ethanol-dependent invasive growth (Figures 4A–C).

The RTG pathway controls expression of genes that function to ameliorate defects in mitochondrial function (Epstein et al., 2001). The activity of the RTG pathway can be assessed by examining the expression of the *CIT2* gene, which is a target of the retrograde pathway (Liao and Butow, 1993; Chelstowska and Butow, 1995; Kos et al., 1995; Jia et al., 1997; Liu and Butow, 1999) that encodes peroxisome citrate synthase (Kim et al., 1986). Ethanol stimulated the activity of a *CIT2-lacZ* transcriptional reporter (Figure 4D) in a manner that was dependent on Rtg2p (Figure 4D). Interestingly, the data indicates that ethanol induces the RTG pathway. One possibility is that nitrogen and ethanol both activate the RTG pathway. The addition of ethanol to cells grown in nitrogen-limiting media showed an additional stimulation (Figure 4D). Thus, nitrogen limitation and ethanol both contribute to RTG pathway activity. Therefore, the mitochondrial retrograde pathway regulates ethanol-inducible filamentous growth.

### Mitochondrial retrograde pathway regulates ethanol-inducible filamentous growth independent of fMAPK, TOR, and HOG pathways

**Figure 5:**
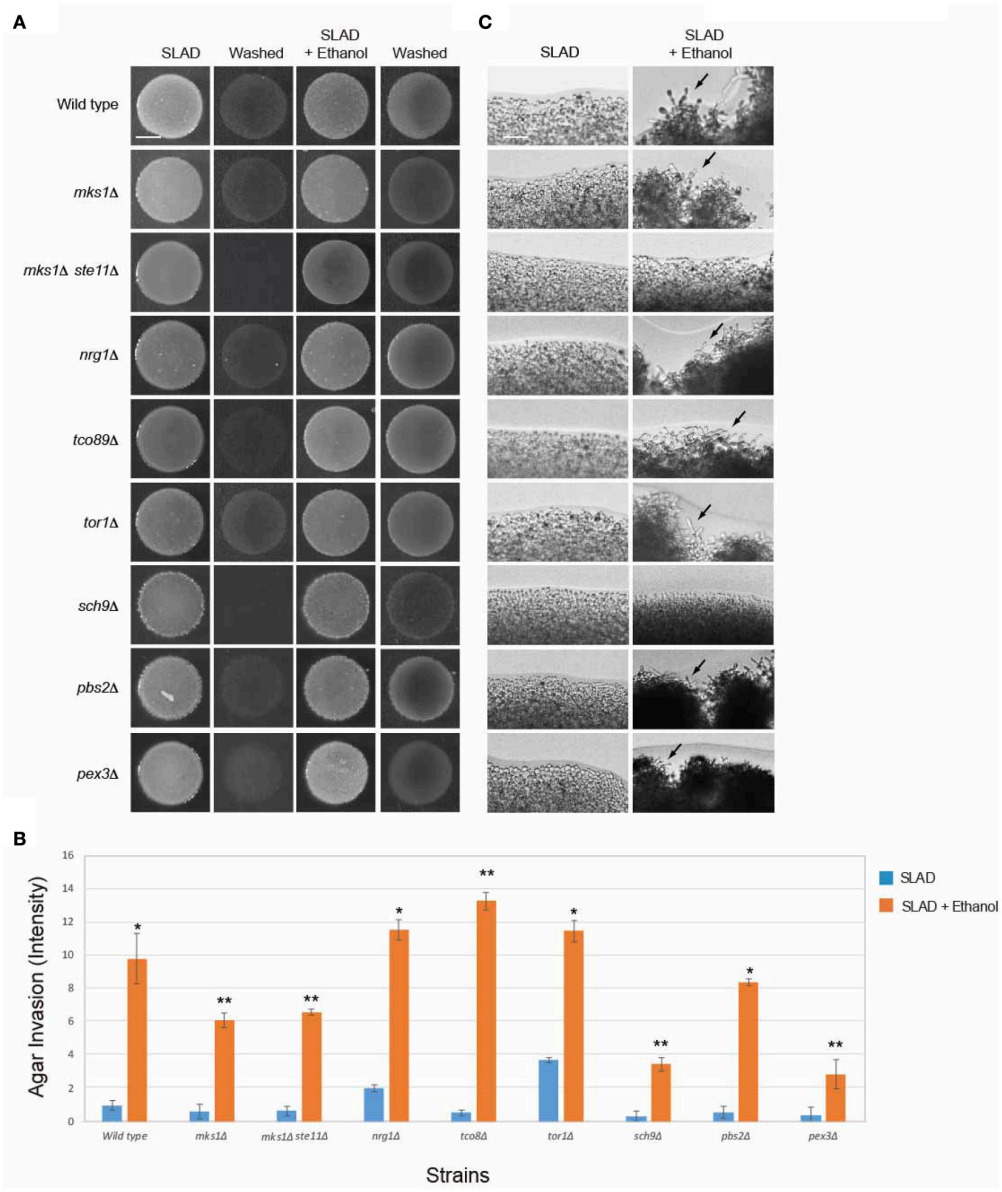
**Role of RTG pathway regulators in controlling ethanol-inducible invasive growth. (A)** Wild-type cells (PC538, Σ1278b *MAT***a** haploid) and the indicated isogenic mutants were spotted onto nitrogen-limiting medium (SLAD) with or without 2% ethanol (v/v). Plates were incubated for 4 days at 30°C, photographed, washed in stream of water, and photographed again. Bar, 5 mm. **(B)** Quantitation of invasive growth in panel **(A)** by densitometry, performed as described in the legend for Figure 1B. Cells were spotted in triplicate, and the average values are shown. Error bars represent the standard difference between experiments. Asterisk denotes a *p* < 0.01 for samples relative to each strain's invasion in SLAD. Double asterisk refers to a *p* < 0.01 for samples relative to each strain's invasion in SLAD compared to wild-type in SLAD with ethanol. **(C)** Colony peripheries from the plates in panel **(A)** were examined by microscopy at 20X magnification. Bar, 50 μm. Arrows mark examples of pseudohyphae.

To define how the RTG pathway connects to the ethanol response, known regulators of that pathway were examined. Mks1p is a negative regulator of multiple pathways, including Rtg2p in the mitochondrial retrograde pathway (Dilova et al., 2004; Ferreira Junior et al., 2005). Mks1p was not required for invasive growth in response to ethanol (Figures 5A–C), which indicates that another negative regulator of the pathway might function in this context. The RTG pathway can regulate the fMAPK pathway (Chavel et al., 2010), as part of a highly coordinated transcriptional sensing and signaling circuit among the pathways that regulate filamentous growth (Borneman et al., 2006; Bharucha et al., 2008; Chavel et al., 2014). We tested whether cells with an up-regulated RTG pathway functioned through fMAPK. An *mks1*Δ *ste11*Δ double mutant, which has an up-regulated retrograde pathway and lacks the MAPKKK for the fMAPK pathway (Ste11p), showed ethanol-inducible invasive growth. This result aligns with the abovementioned results that fMAPK does not regulate ethanol-dependent filamentous growth and indicates that the mitochondrial retrograde pathway does not control filamentation through fMAPK (Figures 5A,B). As shown above, the *mks1*Δ *ste11*Δ double mutant did not show an increase in filamentation at colony peripheries (Figure 5C).

Another major regulator of the mitochondrial retrograde pathway is the TOR pathway, which is a ubiquitous nutrient-regulatory pathway in eukaryotes (Bar-Peled and Sabatini, 2014). TOR plays an important role in nutrient-regulated responses in yeast (Heitman et al., 1991) and is a master regulator of nitrogen control (Beck and Hall, 1999; Cardenas et al., 1999; Bruckner et al., 2011; Kingsbury et al., 2015). TOR signaling also links nitrogen quality to the activity of the Rtg1p and Rtg3p transcription factors (Komeili et al., 2000). TOR specifically regulates the expression of genes encoding RTG pathway components (Crespo et al., 2002; Dilova et al., 2004). We found that the TOR pathway was not required for ethanol-inducible filamentous growth (Figures 5A–C; *tor1*Δ, *tco89*Δ). In addition, the AGC-type kinase Sch9p, which is phosphorylated by and is a major target of TORC1, and which contributes to TORC1-mediated regulation of ribosome biogenesis (Urban et al., 2007; Wei and Zheng, 2009), was not required for ethanol-dependent invasion (*sch9*Δ Figures 5A,B, although it was required for filamentation at colony perimeters Figure 5C). These results may not be entirely surprising, because although TOR and the mitochondrial retrograde pathway are functionally connected, the retrograde response to mitochondrial dysfunction is not dependent on TOR1-dependent regulation of retrograde gene expression (Giannattasio et al., 2005). Therefore, the mitochondrial retrograde pathway controls ethanol-inducible filamentous growth independent of TOR and at least partly independently of Sch9p.

In addition to TOR, the SAP- or p38-type high osmolarity glycerol response (HOG) MAP kinase pathway, which controls the response to osmotic and other stresses (Westfall et al., 2004; Saito, 2010), also regulates the RTG pathway (Ruiz-Roig et al., 2012). The HOG pathway was not required for ethanol-inducible filamentous growth (Figures 5A–C, *pbs2*Δ). Another function of the RTG pathway is to stimulate peroxisome biogenesis in periods of mitochondrial stress (Liao and Butow, 1993; Chelstowska and Butow, 1995; Kos et al., 1995; Epstein et al., 2001). Peroxisomes, which control elements of metabolism and can be regulated by the RTG pathway (Chelstowska and Butow, 1995), may impact ethanol-dependent filamentous growth. A mutant lacking peroxisomes was not required for ethanol-dependent filamentous growth, indicating that this is not the case (Figures 5A–C, *pex3*Δ). However, the *pex3*Δ mutant did show some defect (Figure 5B), and Cit2p, which is a target of RTG, was induced by ethanol (Figure 4D). These proteins regulate the glyoxylate cycle (Jazwinski, 2013) and it is possible that that metabolic pathway plays a role in regulating ethanol-inducible filamentous growth. Therefore, the mitochondrial retrograde pathway regulates ethanol-inducible filamentous growth in a manner that is separate from TOR, fMAPK, and HOG, and partly independent of peroxisome function.

### Regulation of the TCA cycle underlies the role of the mitochondrial retrograde pathway in controlling ethanol-inducible filamentous growth

**Figure 6:**
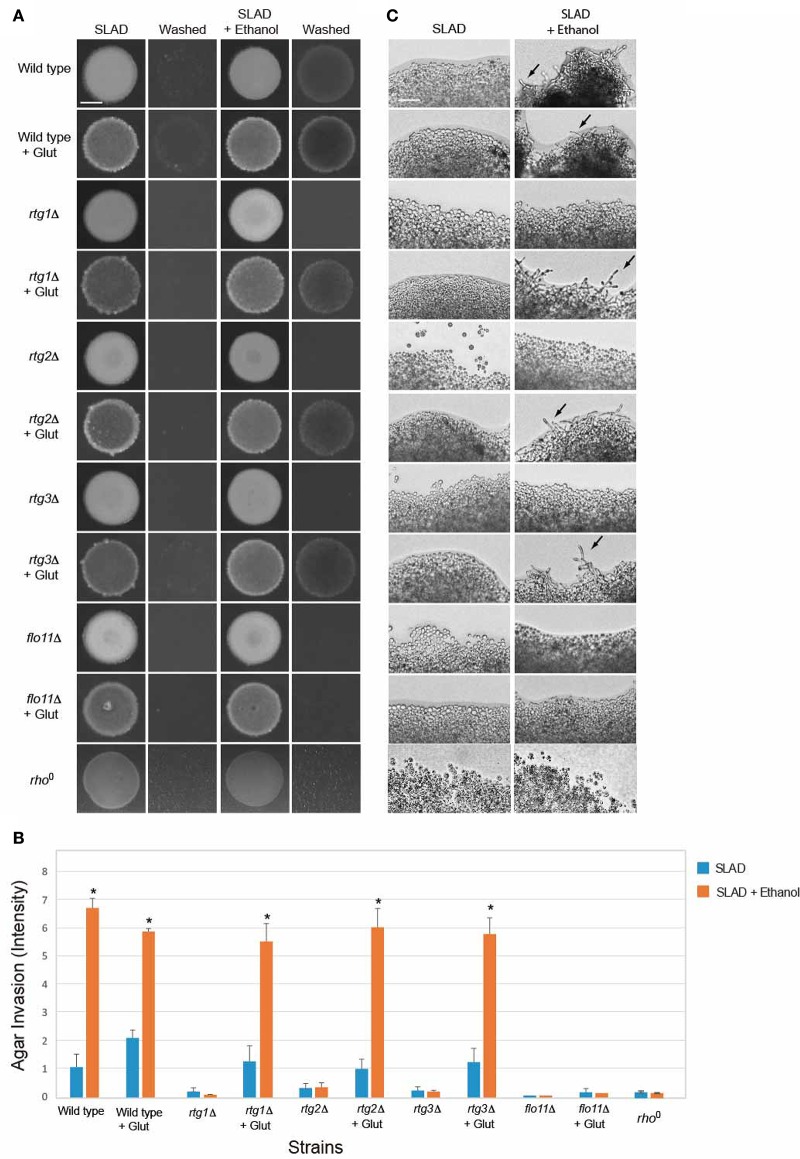
**Impact of glutamate on ethanol-inducible invasive growth defect of ***rtg*** mutants. (A)** Wild-type cells (PC538, Σ1278b *MAT***a** haploid) and the indicated isogenic mutants were spotted onto nitrogen limiting medium (SLAD) with or without 2% ethanol (v/v). Plates were incubated for 4 days at 30°C, photographed, washed in stream of water, and photographed again. Bar, 5 mm. Glutamate was added at a concentration of 200 μM. **(B)** Quantitation of invasive growth in panel **(A)** by densitometry. Cells were spotted in triplicate, and the average values are shown. Error bars represent the standard difference between experiments. Asterisk denotes a *p* < 0.05 for samples relative to each strain's invasion in SLAD. **(C)** Colony peripheries from the plates in panel **(A)** were examined by microscopy at 20X magnification. Bar, 50 μm. Arrows mark examples of pseudohyphae.

The tricarboxylic acid (TCA or citric acid/Krebs) cycle functions through a series of reactions to generate ATP and produce reducing agents necessary for mitochondrial electron transport and energy generation. The TCA cycle is compromised in cells experiencing mitochondrial defects, but flux through the pathway can be maintained by the action of the RTG pathway (Liu and Butow, 1999; Lin et al., 2011), which is a major function of the RTG pathway (Butow and Avadhani, 2004). Glutamate can suppress the requirement for the retrograde pathway in the TCA cycle by increasing metabolic flux (Liu and Butow, 1999). Glutamate suppressed the defect in ethanol-inducible filamentous growth of the *rtg1*Δ, *rtg2*Δ, and *rtg3*Δ mutants (Figures 6A–C). The role of the RTG pathway in regulating ethanol-inducible invasion suggests that mitochondrial respiration is important for ethanol-dependent invasive growth. Consistent with this possibility, *rho*^0^ cells, which lack a functional mitochondria, were defective for ethanol-inducible invasive growth (Figures 6A–C). Thus, one function of the RTG pathway in ethanol-dependent filamentous growth is to stimulate flux through the TCA cycle. Glutamate did not suppress the invasive growth defect of the *flo11*Δ mutant (Figures 6A–C). Given that Flo11p is the main cell adhesion molecule that regulates filamentous growth, these results suggest that glutamate-dependent invasive growth in *rtg* mutants is mediated (in some manner) through Flo11p.

The mitochondrial retrograde pathway has also been shown to regulate deoxyribonucleotide pools by impacting the rate of threonine metabolism (Hartman, 2007). Hydroxyurea induces a cell-cycle delay (Adams and Lindsay, 1967) and reduces the rate of DNA synthesis (Niu et al., 2008), and accordingly triggers a filamentation-like response (Jiang and Kang, 2003). Ethanol may impact threonine levels and DNA synthesis rates and induce retrograde-dependent filamentation. However, hydroxyurea, unlike ethanol, did not cause invasive growth in SLAD medium (Figure S4B). Moreover, the elongated cell morphology induced by hydroxyurea was retrograde-independent (Figure S4C). Therefore, the mitochondrial retrograde pathway probably does not regulate ethanol-dependent filamentous growth by influencing the rate of threonine metabolism.

Several other mutants that are defective in pathways surrounding the TCA cycle, ethanol uptake and metabolism, signaling, and the cell cycle were examined for a role in ethanol-inducible filamentous growth (Figure S5). Most of the mutants examined showed a detectable reduction in ethanol-inducible invasive growth (Figure S5). Two mutants stood out. One lacked Adh2p, which might be expected as that protein catalyzes the conversion of ethanol to acetaldehyde (Bennetzen and Hall, 1982; Young and Pilgrim, 1985; Dickinson et al., 2003). The other lacked Csf1p (Figure S5), a protein that is required for fermentation at low temperatures (Tokai et al., 2000). Notably, the wine yeast P24, which does not invade the agar in SLAD medium, is defective for growth at low temperatures (García-Ríos et al., 2014). Thus, the regulators of ethanol-inducible filamentous growth may encompass a more diverse collection of proteins than has been defined here.

### Ethanol-inducible filamentous growth requires the polarisome and occurs through induction of FLO11 expression

**Figure 7:**
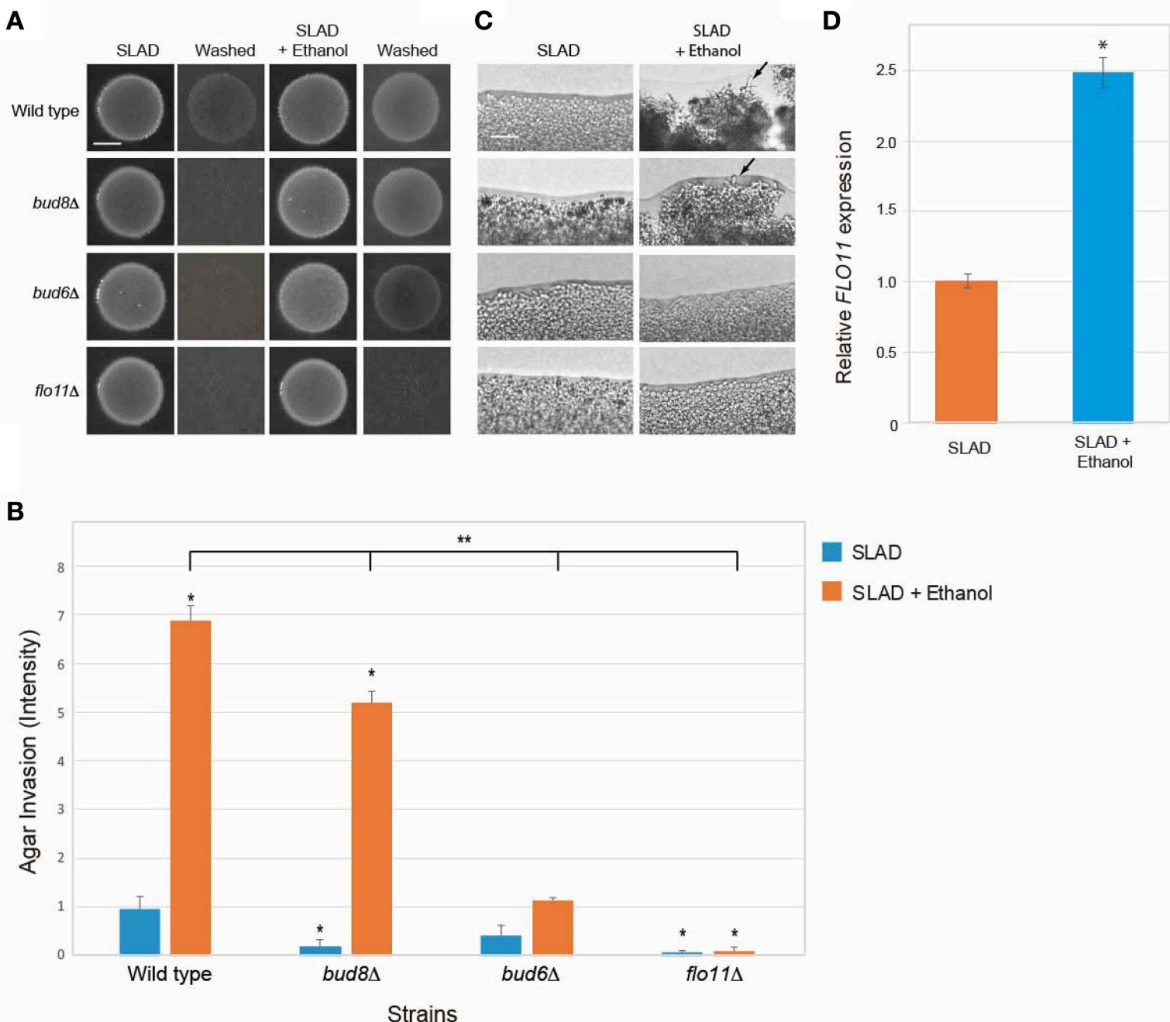
**Requirement for Bud8, Bud6, and Flo11 in mediating ethanol-inducible invasive growth. (A)** Wild-type cells (PC538, Σ1278b *MAT***a** haploid) and the indicated isogenic mutants were spotted onto nitrogen-limiting medium (SLAD) with or without ethanol (2%v/v). Plates were incubated for 4 days at 30°C, photographed, washed in stream of water, and photographed again. Bar, 5 mm. **(B)** Quantitation of invasive growth in panel **(A)** by densitometry, performed as described in the legend for Figure 1B. Asterisk denotes a *p* < 0.01 for samples relative to wild type invasion in SLAD. Double asterisk denotes a *p* < 0.01 for samples relative to wild type invasion in SLAD with ethanol. **(C)** Colony peripheries from the plates in panel **(A)** were examined by microscopy 20X magnification. Bar, 50 μm. **(D)** Ethanol stimulates *FLO11* expression in SLAD medium. Cells were incubated in SLAD (orange bar) or SLAD with ethanol (blue bar). Gene expression was examined by qPCR at time 45 min and normalized to a control transcript (*ACT1*). Error bar represents standard difference between samples. Asterisk denotes a *p* < 0.01.

Filamentous growth involves at least three major regulatory changes. One is an increase in cell length, which is mediated by a delay in the cell cycle (Kron et al., 1994) and by an increase in polarized growth by a Cdc42p-dependent mechanism that involves the polarisome (Cullen and Sprague, 2002). The formin Bni1p (Evangelista et al., 1997) and accessory proteins Bud6p, Pea2p, and Spa2p comprise the polarisome (Amberg et al., 1997; Sagot et al., 2002; Graziano et al., 2011; Tu et al., 2012). Another change is a switch in polarity to distal-unipolar budding that requires the distal-pole landmark Bud8p (Gimeno et al., 1992; Cullen and Sprague, 2002). Bud8p is a distal-pole marker that localizes to the distal pole of the cell (Harkins et al., 2001). The third change, as discussed above, is an increase in adhesion mediated by the cell adhesion molecule Flo11p (Lambrechts et al., 1996; Lo and Dranginis, 1996; Guo et al., 2000). The different aspects of filamentous growth are genetically separable and can be examined by mutants that specifically compromise each aspect of the response (Cullen and Sprague, 2002). Mutants were examined that were specifically defective for polarized growth (*bud6*Δ), polarity reorganization (*bud8*Δ), or cell adhesion (*flo11*Δ). Ethanol-inducible filamentous growth occurred in cells lacking Bud8 (Figures 7A–C, *bud8*Δ), which indicates that ethanol does not function mainly through the switch in polarity. Ethanol-inducible filamentous growth was reduced in cells lacking the polarisome component Bud6p (Figures 7A–C, *bud6*Δ). Thus, ethanol induces filamentous growth by a mechanism that is partly dependent on the increase in polarized growth driven by the polarisome. This is consistent with studies of fusel alcohols, which induce dramatic changes in cell length (Dickinson, 1996; Lorenz et al., 2000). Ethanol-inducible filamentous growth was also dependent on Flo11p (Figures 7A–C, *flo11*Δ). Consistent with this result, ethanol stimulated the expression of the *FLO11* gene (Figure 7D). Therefore, ethanol-inducible filamentous growth, which is controlled by the RTG pathway, requires polarisome function and occurs by a mechanism that involves Flo11p-dependent transcriptional induction.

## Discussion

Filamentous growth in yeast has been mainly studied in one strain background (∑1278b; Gimeno et al., 1992), in part because most laboratory strains have lost filamentation properties due to genetic manipulation in the laboratory (Liu et al., 1996; Dowell et al., 2010; Chin et al., 2012). Although filamentous growth is common among “wild” *S. cerevisiae* strains (Carstens et al., 1998; Sidari et al., 2014), the triggers of filamentous growth have not been extensively characterized in other backgrounds. By examining a collection of wine yeast, we show that most wine strains undergo filamentous growth. The strains also showed a high degree of phenotypic variation. Phenotypic variation is common among individual strains (Dowell et al., 2010) and may not be surprising given that these strains have undergone selection based on flavor, cold-sensitivity, alcohol tolerance, and flocculation (Suzzi et al., 1984; Fleet, 2003; Borneman et al., 2011).

We show here that nitrogen limitation and carbon limitation induce filamentous growth in most strains. This is consistent with previous claims that nitrogen limitation (Gimeno and Fink, 1994) and carbon limitation (Cullen and Sprague, 2000) trigger the filamentation response. We also show that ethanol and fusel alcohols induce filamentous growth. Ethanol (Dickinson, 1994; Lorenz et al., 2000) and fusel alcohols (Dickinson, 1996; Chen and Fink, 2006) are known to stimulate filamentous growth. Fusel alcohols induced filamentous growth under nutrient-replete conditions, and ethanol stimulated filamentous growth under nitrogen-limiting conditions. Ethanol is a by-product of glycolysis, whereas fusel alcohols are by-products of Ehrlich reactions. Thus, the two types of alcohols may provide information about different nutritional states. During alcoholic fermentation, *S. cerevisiae* produces ethanol when it has reached a maximum population density that corresponds with consumption of nitrogen (Beltran et al., 2005). Because nitrogen limitation is itself a trigger for filamentous growth, ethanol may be a coincidence detector of nitrogen levels and TCA compromise. Alternatively, glucose uptake correlates with the rate of the TCA cycle (Heyland et al., 2009). We also identify a potential role for the glyoxylate cycle in regulating ethanol-dependent filamentous growth. Thus, ethanol production may be a readout of nitrogen or glucose availability.

The cellular response to mitochondrial stress is important for biological responses in many systems. Generally speaking, cellular responses to mitochondrial disfunction have been implicated in cancer (Guha and Avadhani, 2013), aging (Friis et al., 2014; da Cunha et al., 2015; Jazwinski, 2015), development (Berkowitz et al., 2016), and inter-organellar homeostasis (Liu and Butow, 2006). Here, we show that the fungal-specific RTG pathway controls ethanol-inducible invasive growth in yeast. Lorenz and Heitman argued that the fMAPK pathway mediates the response to alcohols (Lorenz et al., 2000), and we show that it may play a minor role. Here we establish the RTG pathway as a key pathway in the response. How does the RTG pathway control ethanol-dependent filamentous growth without involving other major filamentation regulatory pathways? One possibility is that the RTG pathway is part of the sensing/signaling mechanism that controls the rate of flux through the TCA cycle (Liu and Butow, 1999; Lin et al., 2011). TCA cycle rate is dependent on carbon and nitrogen levels, which are key inducers of filamentous growth in yeast and other fungal species. Canonical metabolic regulatory pathways that control filamentous growth also control TCA cycle flux including Snf1 (Hedbacker and Carlson, 2008) and TOR (Komeili et al., 2000); thus, TCA cycle activity may be a nexus for monitoring nutritional health.

The connection between TCA cycle flux and filamentous growth may be relevant from the perspective of pathogenecity. TCA cycle flux has been connected to the evolution of pathogenicity in filamentous fungi (Hogan et al., 2015) and apicomplexan parasites (Oppenheim et al., 2014). TCA cycle reprogramming is becoming increasingly tied to developmental transitions in pathogens ranging from *C. albicans* (Askew et al., 2009; Guedouari et al., 2014; Grahl et al., 2015), to *Plasmodium falciparum* (Ke et al., 2015) to *Yersinia pseudotuberculosis* (Bucker et al., 2014). The boost in TCA cycle flux is critical for phagososomal escape of the bacterial pathogen *Francisella* (Ramond et al., 2014). Moreover, the fungal RTG pathway is responsible for evasion of programmed cell death in yeast cells growing on non-repressing carbon sources (Guaragnella et al., 2013). Both the RTG pathway and relief of carbon catabolite repression are required for programmed cell death resistance. Evasion of programmed cell death and filamentous growth may be two hallmarks that fungi must acquire to become pathogenic. Our study therefore connects TCA cycle flux, as regulated by the RTG pathway, to an aspect of filamentous growth. Perhaps TCA flux controls developmental and morphogenetic responses in other eukaryotic systems.

## Author contributions

BG designed and performed experiments. GB designed experiments. AM designed experiments. MJT designed experiments. PC helped with experimental design and writing the paper.

## Funding

PC is supported from grants from the NIH (GM098629 and DE022720). The work was supported by the Ministry of Economy and Competitiveness, Spain (Grant no. AGL2013-47300-C3). BG is grateful to the pre-doctoral fellowship from the University Rovira i Virgili and the Oenological Biotechnology research group for a mobility grant.

### Conflict of interest statement

The authors declare that the research was conducted in the absence of any commercial or financial relationships that could be construed as a potential conflict of interest.

## Abbreviations

AMPK: AMP-dependent protein kinase
DIC: differential-interference-contrast
fMAPK: filamentous growth mitogen activated protein kinase
HOG: high osmolarity glycerol pathway
MM: minimal medium
OD: optical density
PWA: plate-washing assay
PKA: protein kinase A
qPCR: quantitative polymerase chain reaction
SAD: synthetic medium with ammonium and dextrose
SALG: synthetic medium with ammonium and low glucose
SLAD: synthetic medium with dextrose and low-ammonium
TOR: target of rapamycin
TCA: tricarboxylic acid
Trp-OH: tryptophol
v/v: volume-to-volume percent
YNB: yeast nitrogen base
YPD: yeast peptone dextrose.

## References

[B1] Abdullah U. Cullen P. J. (2009). The tRNA modification complex elongator regulates the Cdc42-dependent mitogen-activated protein kinase pathway that controls filamentous growth in yeast. Eukaryot. Cell 8, 1362–1372. 10.1128/EC.00015-09 19633267 PMC2747823

[B2] Adams R. L. Lindsay J. G. (1967). Hydroxyurea reversal of inhibition and use as a cell-synchronizing agent. J. Biol. Chem. 242, 1314–1317. 6023572

[B3] Adhikari H. Cullen P. J. (2014). Metabolic respiration induces AMPK- and Ire1p-dependent activation of the p38-Type HOG MAPK Pathway. PLoS Genet. 10:e1004734. 10.1371/journal.pgen.1004734 25356552 PMC4214603

[B4] Adhikari H. Vadaie N. Chow J. Caccamise L. M. Chavel C. A. Li B.. (2015). Role of the unfolded protein response in regulating the mucin-dependent filamentous-growth mitogen-activated protein kinase pathway. Mol. Cell. Biol. 35, 1414–1432. 10.1128/MCB.01501-14 25666509 PMC4372694

[B5] Albuquerque P. Casadevall A. (2012). Quorum sensing in fungi–a review. Med. Mycol. 50, 337–345. 10.3109/13693786.2011.652201 22268493 PMC4294699

[B6] Alper H. Moxley J. Nevoigt E. Fink G. R. Stephanopoulos G. (2006). Engineering yeast transcription machinery for improved ethanol tolerance and production. Science 314, 1565–1568. 10.1126/science.1131969 17158319

[B7] Amberg D. C. Zahner J. E. Mulholland J. W. Pringle J. R. Botstein D. (1997). Aip3p/Bud6p, a yeast actin-interacting protein that is involved in morphogenesis and the selection of bipolar budding sites. Mol. Biol. Cell 8, 729–753. 10.1091/mbc.8.4.729 9247651 PMC276122

[B8] Argueso J. L. Carazzolle M. F. Mieczkowski P. A. Duarte F. M. Netto O. V. Missawa S. K.. (2009). Genome structure of a *Saccharomyces cerevisiae* strain widely used in bioethanol production. Genome Res. 19, 2258–2270. 10.1101/gr.091777.109 19812109 PMC2792172

[B9] Askew C. Sellam A. Epp E. Hogues H. Mullick A. Nantel A.. (2009). Transcriptional regulation of carbohydrate metabolism in the human pathogen *Candida albicans*. PLoS Pathog. 5:e1000612. 10.1371/journal.ppat.1000612 19816560 PMC2749448

[B10] Aun A. Tamm T. Sedman J. (2013). Dysfunctional mitochondria modulate cAMP-PKA signaling and filamentous and invasive growth of *Saccharomyces cerevisiae*. Genetics 193, 467–481. 10.1534/genetics.112.147389 23172851 PMC3567737

[B11] Avbelj M. Zupan J. Kranjc L. Raspor P. (2015). Quorum-sensing kinetics in *Saccharomyces cerevisiae*: a symphony of ARO genes and aromatic alcohols. J. Agric. Food Chem. 63, 8544–8550. 10.1021/acs.jafc.5b03400 26367540

[B12] Bar-Peled L. Sabatini D. M. (2014). Regulation of mTORC1 by amino acids. Trends Cell Biol. 24, 400–406. 10.1016/j.tcb.2014.03.003 24698685 PMC4074565

[B13] Barrales R. R. Jimenez J. Ibeas J. I. (2008). Identification of novel activation mechanisms for FLO11 regulation in *Saccharomyces cerevisiae*. Genetics 178, 145–156. 10.1534/genetics.107.081315 18202364 PMC2206066

[B14] Beck T. Hall M. N. (1999). The TOR signalling pathway controls nuclear localization of nutrient- regulated transcription factors. Nature 402, 689–692. 10.1038/45287 10604478

[B15] Beltran G. Esteve-Zarzoso B. Rozes N. Mas A. Guillamon J. M. (2005). Influence of the timing of nitrogen additions during synthetic grape must fermentations on fermentation kinetics and nitrogen consumption. J. Agric. Food Chem. 53, 996–1002. 10.1021/jf0487001 15713011

[B16] Beltran G. Novo M. Guillamon J. M. Mas A. Rozes N. (2008). Effect of fermentation temperature and culture media on the yeast lipid composition and wine volatile compounds. Int. J. Food Microbiol. 121, 169–177. 10.1016/j.ijfoodmicro.2007.11.030 18068842

[B17] Beltran G. Novo M. Rozes N. Mas A. Guillamon J. M. (2004). Nitrogen catabolite repression in *Saccharomyces cerevisiae* during wine fermentations. FEMS Yeast Res. 4, 625–632. 10.1016/j.femsyr.2003.12.004 15040951

[B18] Bennetzen J. L. Hall B. D. (1982). The primary structure of the *Saccharomyces cerevisiae* gene for alcohol dehydrogenase. J. Biol. Chem. 257, 3018–3025. 6277922

[B19] Berkowitz O. De Clercq I. Van Breusegem F. Whelan J. (2016). Interaction between hormonal and mitochondrial signalling during growth, development and in plant defence responses. Plant Cell Environ. 39, 1127–1139. 10.1111/pce.12712 26763171

[B20] Bharucha N. Ma J. Dobry C. J. Lawson S. K. Yang Z. Kumar A. (2008). Analysis of the yeast kinome reveals a network of regulated protein localization during filamentous growth. Mol. Biol. Cell 19, 2708–2717. 10.1091/mbc.E07-11-1199 18417610 PMC2441683

[B21] Bojsen R. K. Andersen K. S. Regenberg B. (2012). *Saccharomyces cerevisiae*–a model to uncover molecular mechanisms for yeast biofilm biology. FEMS Immunol. Med. Microbiol. 65, 169–182. 10.1111/j.1574-695X.2012.00943.x 22332975

[B22] Borneman A. R. Desany B. A. Riches D. Affourtit J. P. Forgan A. H. Pretorius I. S.. (2011). Whole-genome comparison reveals novel genetic elements that characterize the genome of industrial strains of *Saccharomyces cerevisiae*. PLoS Genet. 7:e1001287. 10.1371/journal.pgen.1001287 21304888 PMC3033381

[B23] Borneman A. R. Desany B. A. Riches D. Affourtit J. P. Forgan A. H. Pretorius I. S.. (2012). The genome sequence of the wine yeast VIN7 reveals an allotriploid hybrid genome with *Saccharomyces cerevisiae* and *Saccharomyces kudriavzevii* origins. FEMS Yeast Res. 12, 88–96. 10.1111/j.1567-1364.2011.00773.x 22136070

[B24] Borneman A. R. Forgan A. H. Pretorius I. S. Chambers P. J. (2008). Comparative genome analysis of a *Saccharomyces cerevisiae* wine strain. FEMS Yeast Res. 8, 1185–1195. 10.1111/j.1567-1364.2008.00434.x 18778279

[B25] Borneman A. R. Leigh-Bell J. A. Yu H. Bertone P. Gerstein M. Snyder M. (2006). Target hub proteins serve as master regulators of development in yeast. Genes Dev. 20, 435–448. 10.1101/gad.1389306 16449570 PMC1369046

[B26] Borneman A. R. Pretorius I. S. (2015). Genomic insights into the *Saccharomyces sensu stricto* complex. Genetics 199, 281–291. 10.1534/genetics.114.173633 25657346 PMC4317643

[B27] Bruckner S. Kern S. Birke R. Saugar I. Ulrich H. D. Mösch H. U. (2011). The TEA transcription factor Tec1 links TOR and MAPK pathways to coordinate yeast development. Genetics 189, 479–494. 10.1534/genetics.111.133629 21840851 PMC3189810

[B28] Bucker R. Heroven A. K. Becker J. Dersch P. Wittmann C. (2014). The pyruvate-tricarboxylic acid cycle node: a focal point of virulence control in the enteric pathogen *Yersinia pseudotuberculosis*. J. Biol. Chem. 289, 30114–30132. 10.1074/jbc.M114.581348 25164818 PMC4208018

[B29] Butow R. A. Avadhani N. G. (2004). Mitochondrial signaling: the retrograde response. Mol. Cell 14, 1–15. 10.1016/S1097-2765(04)00179-0 15068799

[B30] Cardenas M. E. Cutler N. S. Lorenz M. C. Di Como C. J. Heitman J. (1999). The TOR signaling cascade regulates gene expression in response to nutrients. Genes Dev. 13, 3271–3279. 10.1101/gad.13.24.3271 10617575 PMC317202

[B31] Carrozza M. J. Florens L. Swanson S. K. Shia W. J. Anderson S. Yates J.. (2005). Stable incorporation of sequence specific repressors Ash1 and Ume6 into the Rpd3L complex. Biochim. Biophys. Acta 1731, 77–87. 10.1016/j.bbaexp.2005.09.005 16314178

[B32] Carstens E. Lambrechts M. G. Pretorius I. S. (1998). Flocculation, pseudohyphal development and invasive growth in commercial wine yeast strains. *S*. Afr. J. Enol. Vitc. 19, 52–61.

[B33] Celenza J. L. Carlson M. (1989). Mutational analysis of the *Saccharomyces cerevisiae* SNF1 protein kinase and evidence for functional interaction with the SNF4 protein. Mol. Cell. Biol. 9, 5034–5044. 10.1128/MCB.9.11.5034 2557546 PMC363655

[B34] Chavel C. A. Caccamise L. M. Li B. Cullen P. J. (2014). Global regulation of a differentiation MAPK pathway in yeast. Genetics 198, 1309–1328. 10.1534/genetics.114.168252 25189875 PMC4224168

[B35] Chavel C. A. Dionne H. M. Birkaya B. Joshi J. Cullen P. J. (2010). Multiple signals converge on a differentiation MAPK pathway. PLoS Genet. 6:e1000883. 10.1371/journal.pgen.1000883 20333241 PMC2841618

[B36] Chelstowska A. Butow R. A. (1995). RTG genes in yeast that function in communication between mitochondria and the nucleus are also required for expression of genes encoding peroxisomal proteins. J. Biol. Chem. 270, 18141–18146. 10.1074/jbc.270.30.18141 7629125

[B37] Chen H. Fink G. R. (2006). Feedback control of morphogenesis in fungi by aromatic alcohols. Genes Dev. 20, 1150–1161. 10.1101/gad.1411806 16618799 PMC1472474

[B38] Chen H. Fujita M. Feng Q. Clardy J. Fink G. R. (2004). Tyrosol is a quorum-sensing molecule in *Candida albicans*. Proc. Natl. Acad. Sci. U.S.A. 101, 5048–5052. 10.1073/pnas.0401416101 15051880 PMC387371

[B39] Chin B. L. Ryan O. Lewitter F. Boone C. Fink G. R. (2012). Genetic variation in *Saccharomyces cerevisiae*: circuit diversification in a signal transduction network. Genetics 192, 1523–1532. 10.1534/genetics.112.145573 23051644 PMC3512157

[B40] Colombo S. Ma P. Cauwenberg L. Winderickx J. Crauwels M. Teunissen A.. (1998). Involvement of distinct G-proteins, Gpa2 and Ras, in glucose- and intracellular acidification-induced cAMP signalling in the yeast *Saccharomyces cerevisiae*. EMBO J. 17, 3326–3341. 10.1093/emboj/17.12.3326 9628870 PMC1170671

[B41] Crespo J. L. Powers T. Fowler B. Hall M. N. (2002). The TOR-controlled transcription activators GLN3, RTG1, and RTG3 are regulated in response to intracellular levels of glutamine. Proc. Natl. Acad. Sci. U.S.A. 99, 6784–6789. 10.1073/pnas.102687599 11997479 PMC124480

[B42] Cullen P. J. Sabbagh W. Jr. Graham E. Irick M. M. van Olden E. K. Neal C.. (2004). A signaling mucin at the head of the Cdc42- and MAPK-dependent filamentous growth pathway in yeast. Genes Dev. 18, 1695–1708. 10.1101/gad.1178604 15256499 PMC478191

[B43] Cullen P. J. Sprague G. F. Jr. (2000). Glucose depletion causes haploid invasive growth in yeast. Proc. Natl. Acad. Sci. U.S.A. 97, 13619–13624. 10.1073/pnas.240345197 11095711 PMC17625

[B44] Cullen P. J. Sprague G. F. Jr. (2002). The roles of bud-site-selection proteins during haploid invasive growth in yeast. Mol. Biol. Cell 13, 2990–3004. 10.1091/mbc.E02-03-0151 12221111 PMC124138

[B45] da Cunha F. M. Torelli N. Q. Kowaltowski A. J. (2015). Mitochondrial retrograde signaling: triggers, pathways, and outcomes. Oxid. Med. Cell. Longev. 2015:482582. 10.1155/2015/482582 26583058 PMC4637108

[B46] Dickinson J. R. (1994). Irreversible formation of pseudohyphae by haploid *Saccharomyces cerevisiae*. FEMS Microbiol. Lett. 119, 99–103. 10.1111/j.1574-6968.1994.tb06874.x 8039677

[B47] Dickinson J. R. (1996). “Fusel” alcohols induce hyphal-like extensions and pseudohyphal formation in yeasts. Microbiology 142(Pt 6), 1391–1397. 10.1099/13500872-142-6-1391 8704979

[B48] Dickinson J. R. Salgado L. E. Hewlins M. J. (2003). The catabolism of amino acids to long chain and complex alcohols in *Saccharomyces cerevisiae*. J. Biol. Chem. 278, 8028–8034. 10.1074/jbc.M211914200 12499363

[B49] Dilova I. Aronova S. Chen J. C. Powers T. (2004). Tor signaling and nutrient-based signals converge on Mks1p phosphorylation to regulate expression of Rtg1.Rtg3p-dependent target genes. J. Biol. Chem. 279, 46527–46535. 10.1074/jbc.M409012200 15326168

[B50] Dowell R. D. Ryan O. Jansen A. Cheung D. Agarwala S. Danford T.. (2010). Genotype to phenotype: a complex problem. Science 328, 469. 10.1126/science.1189015 20413493 PMC4412269

[B51] Epstein C. B. Waddle J. A. Hale W. IV. Davé V. Thornton J.. (2001). Genome-wide responses to mitochondrial dysfunction. Mol. Biol. Cell 12, 297–308. 10.1091/mbc.12.2.297 11179416 PMC30944

[B52] Evangelista M. Blundell K. Longtine M. S. Chow C. J. Adames N. Pringle J. R.. (1997). Bni1p, a yeast formin linking cdc42p and the actin cytoskeleton during polarized morphogenesis. Science 276, 118–122. 10.1126/science.276.5309.118 9082982

[B53] Ferreira Junior J. R. Spirek M. Liu Z. Butow R. A. (2005). Interaction between Rtg2p and Mks1p in the regulation of the RTG pathway of *Saccharomyces cerevisiae*. Gene 354, 2–8. 10.1016/j.gene.2005.03.048 15967597

[B54] Fleet G. H. (2003). Yeast interactions and wine flavour. Int. J. Food Microbiol. 86, 11–22. 10.1016/S0168-1605(03)00245-9 12892919

[B55] Fleet G. H. Heard G. M. (1993). Yeasts: growth during fermentation, in Wine Microbiology and Biotechnology, ed. Fleet G. H. (Chur: Hardwood Academic Publishers), 27–54.

[B56] Friis R. M. Glaves J. P. Huan T. Li L. Sykes B. D. Schultz M. C. (2014). Rewiring AMPK and mitochondrial retrograde signaling for metabolic control of aging and histone acetylation in respiratory-defective cells. Cell Rep. 7, 565–574. 10.1016/j.celrep.2014.03.029 24726357

[B57] García-Ríos E. López-Malo M. Guillamón J. M. (2014). Global phenotypic and genomic comparison of two *Saccharomyces cerevisiae* wine strains reveals a novel role of the sulfur assimilation pathway in adaptation at low temperature fermentations. BMC Genomics 15:1059. 10.1186/1471-2164-15-1059 25471357 PMC4265444

[B58] Ghosh S. Kebaara B. W. Atkin A. L. Nickerson K. W. (2008). Regulation of aromatic alcohol production in *Candida albicans*. Appl. Environ. Microbiol. 74, 7211–7218. 10.1128/AEM.01614-08 18836025 PMC2592902

[B59] Giannattasio S. Liu Z. Thornton J. Butow R. A. (2005). Retrograde response to mitochondrial dysfunction is separable from TOR1/2 regulation of retrograde gene expression. J. Biol. Chem. 280, 42528–42535. 10.1074/jbc.M509187200 16253991

[B60] Gimeno C. J. Fink G. R. (1994). Induction of pseudohyphal growth by overexpression of PHD1, a *Saccharomyces cerevisiae* gene related to transcriptional regulators of fungal development. Mol. Cell. Biol. 14, 2100–2112. 10.1128/MCB.14.3.2100 8114741 PMC358570

[B61] Gimeno C. J. Ljungdahl P. O. Styles C. A. Fink G. R. (1992). Unipolar cell divisions in the yeast *S. cerevisiae* lead to filamentous growth: regulation by starvation and RAS. Cell 68, 1077–1090. 10.1016/0092-8674(92)90079-R 1547504

[B62] Goffeau A. Barrell B. G. Bussey H. Davis R. W. Dujon B. Feldmann H. (1996). Life with 6000 genes. Science 274, 546, 563–547. 10.1126/science.274.5287.546 8849441

[B63] Goldstein A. L. McCusker J. H. (1999). Three new dominant drug resistance cassettes for gene disruption in *Saccharomyces cerevisiae*. Yeast 15, 1541–1553. 10514571 10.1002/(SICI)1097-0061(199910)15:14<1541::AID-YEA476>3.0.CO;2-K

[B64] Grahl N. Demers E. G. Lindsay A. K. Harty C. E. Willger S. D. Piispanen A. E.. (2015). Mitochondrial activity and Cyr1 are key regulators of Ras1 activation of *C. albicans* virulence pathways. PLoS Pathog 11:e1005133. 10.1371/journal.ppat.1005133 26317337 PMC4552728

[B65] Graziano B. R. DuPage A. G. Michelot A. Breitsprecher D. Moseley J. B. Sagot I.. (2011). Mechanism and cellular function of Bud6 as an actin nucleation-promoting factor. Mol. Biol. Cell 22, 4016–4028. 10.1091/mbc.E11-05-0404 21880892 PMC3204064

[B66] Guaragnella N. Zdralevic M. Lattanzio P. Marzulli D. Pracheil T. Liu Z.. (2013). Yeast growth in raffinose results in resistance to acetic-acid induced programmed cell death mostly due to the activation of the mitochondrial retrograde pathway. Biochim. Biophys. Acta 1833, 2765–2774. 10.1016/j.bbamcr.2013.07.017 23906793

[B67] Guedouari H. Gergondey R. Bourdais A. Vanparis O. Bulteau A. L. Camadro J. M.. (2014). Changes in glutathione-dependent redox status and mitochondrial energetic strategies are part of the adaptive response during the filamentation process in *Candida albicans*. Biochim. Biophys. Acta 1842, 1855–1869. 10.1016/j.bbadis.2014.07.006 25018088

[B68] Guha M. Avadhani N. G. (2013). Mitochondrial retrograde signaling at the crossroads of tumor bioenergetics, genetics and epigenetics. Mitochondrion 13, 577–591. 10.1016/j.mito.2013.08.007 24004957 PMC3832239

[B69] Guo B. Styles C. A. Feng Q. Fink G. R. (2000). A Saccharomyces gene family involved in invasive growth, cell-cell adhesion, and mating. Proc. Natl. Acad. Sci. U.S.A. 97, 12158–12163. 10.1073/pnas.220420397 11027318 PMC17311

[B70] Harkins H. A. Page N. Schenkman L. R. De Virgilio C. Shaw S.. (2001). Bud8p and Bud9p, proteins that may mark the sites for bipolar budding in yeast. Mol. Biol. Cell 12, 2497–2518. 10.1091/mbc.12.8.2497 11514631 PMC58609

[B71] Hartman J. L. T. (2007). Buffering of deoxyribonucleotide pool homeostasis by threonine metabolism. Proc. Natl. Acad. Sci. U.S.A. 104, 11700–11705. 10.1073/pnas.0705212104 17606896 PMC1913885

[B72] Hazelwood L. A. Daran J. M. van Maris A. J. Pronk J. T. Dickinson J. R. (2008). The Ehrlich pathway for fusel alcohol production: a century of research on *Saccharomyces cerevisiae* metabolism. Appl. Environ. Microbiol. 74, 2259–2266. 10.1128/AEM.02625-07 18281432 PMC2293160

[B73] Hedbacker K. Carlson M. (2008). SNF1/AMPK pathways in yeast. Front. Biosci. 13, 2408–2420. 10.2741/2854 17981722 PMC2685184

[B74] Heitman J. Movva N. R. Hall M. N. (1991). Targets for cell cycle arrest by the immunosuppressant rapamycin in yeast. Science 253, 905–909. 10.1126/science.1715094 1715094

[B75] Heyland J. Fu J. Blank L. M. (2009). Correlation between TCA cycle flux and glucose uptake rate during respiro-fermentative growth of *Saccharomyces cerevisiae*. Microbiology 155, 3827–3837. 10.1099/mic.0.030213-0 19684065

[B76] Hlavacek O. Kucerova H. Harant K. Palkova Z. Vachova L. (2009). Putative role for ABC multidrug exporters in yeast quorum sensing. FEBS Lett. 583, 1107–1113. 10.1016/j.febslet.2009.02.030 19250938

[B77] Hogan G. J. Brown P. O. Herschlag D. (2015). Evolutionary Conservation and diversification of Puf RNA binding proteins and their mRNA targets. PLoS Biol. 13:e1002307. 10.1371/journal.pbio.1002307 26587879 PMC4654594

[B78] Huang D. Friesen H. Andrews B. (2007). Pho85, a multifunctional cyclin-dependent protein kinase in budding yeast. Mol. Microbiol. 66, 303–314. 10.1111/j.1365-2958.2007.05914.x 17850263

[B79] Huang D. Moffat J. Andrews B. (2002). Dissection of a complex phenotype by functional genomics reveals roles for the yeast cyclin-dependent protein kinase Pho85 in stress adaptation and cell integrity. Mol. Cell. Biol. 22, 5076–5088. 10.1128/MCB.22.14.5076-5088.2002 12077337 PMC139770

[B80] Jazwinski S. M. (2013). The retrograde response: when mitochondrial quality control is not enough. Biochim. Biophys. Acta 1833, 400–409. 10.1016/j.bbamcr.2012.02.010 22374136 PMC3389569

[B81] Jazwinski S. M. (2015). Mitochondria to nucleus signaling and the role of ceramide in its integration into the suite of cell quality control processes during aging. Ageing Res. Rev. 23, 67–74. 10.1016/j.arr.2014.12.007 25555678 PMC4480153

[B82] Jia Y. Rothermel B. Thornton J. Butow R. A. (1997). A basic helix-loop-helix-leucine zipper transcription complex in yeast functions in a signaling pathway from mitochondria to the nucleus. Mol. Cell. Biol. 17, 1110–1117. 10.1128/MCB.17.3.1110 9032238 PMC231836

[B83] Jiang Y. W. Kang C. M. (2003). Induction of *S. cerevisiae* filamentous differentiation by slowed DNA synthesis involves Mec1, Rad53 and Swe1 checkpoint proteins. Mol. Biol. Cell 14, 5116–5124. 10.1091/mbc.E03-06-0375 14565980 PMC284813

[B84] Jin R. Dobry C. J. McCown P. J. Kumar A. (2008). Large-scale analysis of yeast filamentous growth by systematic gene disruption and overexpression. Mol. Biol. Cell 19, 284–296. 10.1091/mbc.E07-05-0519 17989363 PMC2174193

[B85] Karunanithi S. Cullen P. J. (2012). The filamentous growth MAPK pathway responds to glucose starvation through the Mig1/2 transcriptional repressors in *Saccharomyces cerevisiae*. Genetics 192, 869–887. 10.1534/genetics.112.142661 22904036 PMC3522164

[B86] Ke H. Lewis I. A. Morrisey J. M. McLean K. J. Ganesan S. M. Painter H. J.. (2015). Genetic investigation of tricarboxylic acid metabolism during the *Plasmodium falciparum* life cycle. Cell Rep. 11, 164–174. 10.1016/j.celrep.2015.03.011 25843709 PMC4394047

[B87] Kim K. S. Rosenkrantz M. S. Guarente L. (1986). *Saccharomyces cerevisiae* contains two functional citrate synthase genes. Mol. Cell. Biol. 6, 1936–1942. 10.1128/MCB.6.6.1936 3023912 PMC367731

[B88] Kingsbury J. M. Sen N. D. Cardenas M. E. (2015). Branched-Chain Aminotransferases Control TORC1 Signaling in *Saccharomyces cerevisiae*. PLoS Genet. 11:e1005714. 10.1371/journal.pgen.1005714 26659116 PMC4684349

[B89] Kleine T. Leister D. (2016). Retrograde signaling: organelles go networking. Biochim. Biophys. Acta 1857, 1313–1325. 10.1016/j.bbabio.2016.03.017 26997501

[B90] Komeili A. Wedaman K. P. O'Shea E. K. Powers T. (2000). Mechanism of metabolic control. Target of rapamycin signaling links nitrogen quality to the activity of the Rtg1 and Rtg3 transcription factors. J. Cell Biol. 151, 863–878. 10.1083/jcb.151.4.863 11076970 PMC2169436

[B91] Kos W. Kal A. J. van Wilpe S. Tabak H. F. (1995). Expression of genes encoding peroxisomal proteins in *Saccharomyces cerevisiae* is regulated by different circuits of transcriptional control. Biochim. Biophys. Acta 1264, 79–86. 10.1016/0167-4781(95)00127-3 7578261

[B92] Krogan N. J. Greenblatt J. F. (2001). Characterization of a six-subunit Holo-Elongator complex required for the regulated expression of a group of genes in *Saccharomyces cerevisiae*. Mol. Cell. Biol. 21, 8203–8212. 10.1128/MCB.21.23.8203-8212.2001 11689709 PMC99985

[B93] Kron S. J. Styles C. A. Fink G. R. (1994). Symmetric cell division in pseudohyphae of the yeast *Saccharomyces cerevisiae*. Mol. Biol. Cell 5, 1003–1022. 10.1091/mbc.5.9.1003 7841518 PMC301123

[B94] Kruppa M. (2008). Quorum sensing and *Candida albicans*. Mycoses. 52, 1–10. 10.1111/j.1439-0507.2008.01626.x 18983434

[B95] Kuchin S. Vyas V. K. Carlson M. (2002). Snf1 protein kinase and the repressors Nrg1 and Nrg2 regulate FLO11, haploid invasive growth, and diploid pseudohyphal differentiation. Mol. Cell. Biol. 22, 3994–4000. 10.1128/MCB.22.12.3994-4000.2002 12024013 PMC133850

[B96] Lamb T. M. Mitchell A. P. (2003). The transcription factor Rim101p governs ion tolerance and cell differentiation by direct repression of the regulatory genes NRG1 and SMP1 in *Saccharomyces cerevisiae*. Mol. Cell. Biol. 23, 677–686. 10.1128/MCB.23.2.677-686.2003 12509465 PMC151549

[B97] Lamb T. M. Xu W. Diamond A. Mitchell A. P. (2001). Alkaline response genes of *Saccharomyces cerevisiae* and their relationship to the RIM101 pathway. J. Biol. Chem. 276, 1850–1856. 10.1074/jbc.M008381200 11050096

[B98] Lambrechts M. G. Bauer F. F. Marmur J. Pretorius I. S. (1996). Muc1, a mucin-like protein that is regulated by Mss10, is critical for pseudohyphal differentiation in yeast. Proc. Natl. Acad. Sci. U.S.A. 93, 8419–8424. 10.1073/pnas.93.16.8419 8710886 PMC38686

[B99] Langford M. L. Hargarten J. C. Patefield K. D. Marta E. Blankenship J. R. Fanning S.. (2013). *Candida albicans* Czf1 and Efg1 coordinate the response to farnesol during quorum sensing, white-opaque thermal dimorphism, and cell death. Eukaryot. Cell 12, 1281–1292. 10.1128/EC.00311-12 23873867 PMC3811573

[B100] Lengeler K. B. Davidson R. C. D'Souza C. Harashima T. Shen W. C. Wang P.. (2000). Signal transduction cascades regulating fungal development and virulence. Microbiol. Mol. Biol. Rev. 64, 746–785. 10.1128/MMBR.64.4.746-785.2000 11104818 PMC99013

[B101] Lesage P. Yang X. Carlson M. (1996). Yeast SNF1 protein kinase interacts with SIP4, a C6 zinc cluster transcriptional activator: a new role for SNF1 in the glucose response. Mol. Cell. Biol. 16, 1921–1928. 10.1128/MCB.16.5.1921 8628258 PMC231179

[B102] Li L. Wright S. J. Krystofova S. Park G. Borkovich K. A. (2007). Heterotrimeric G protein signaling in filamentous fungi. Annu. Rev. Microbiol. 61, 423–452. 10.1146/annurev.micro.61.080706.093432 17506673

[B103] Liao X. Butow R. A. (1993). RTG1 and RTG2: two yeast genes required for a novel path of communication from mitochondria to the nucleus. Cell 72, 61–71. 10.1016/0092-8674(93)90050-Z 8422683

[B104] Lin A. P. Anderson S. L. Minard K. I. McAlister-Henn L. (2011). Effects of excess succinate and retrograde control of metabolite accumulation in yeast tricarboxylic cycle mutants. J. Biol. Chem. 286, 33737–33746. 10.1074/jbc.M111.266890 21841001 PMC3190802

[B105] Liti G. Carter D. M. Moses A. M. Warringer J. Parts L. James S. A.. (2009). Population genomics of domestic and wild yeasts. Nature 458, 337–341. 10.1038/nature07743 19212322 PMC2659681

[B106] Liu H. Styles C. A. Fink G. R. (1993). Elements of the yeast pheromone response pathway required for filamentous growth of diploids. Science 262, 1741–1744. 8259520 10.1126/science.8259520

[B107] Liu H. Styles C. A. Fink G. R. (1996). *Saccharomyces cerevisiae* S288C has a mutation in FLO8, a gene required for filamentous growth. Genetics 144, 967–978. 8913742 10.1093/genetics/144.3.967 PMC1207636

[B108] Liu Z. Butow R. A. (1999). A transcriptional switch in the expression of yeast tricarboxylic acid cycle genes in response to a reduction or loss of respiratory function. Mol. Cell. Biol. 19, 6720–6728. 10.1128/MCB.19.10.6720 10490611 PMC84662

[B109] Liu Z. Butow R. A. (2006). Mitochondrial retrograde signaling. Annu. Rev. Genet. 40, 159–185. 10.1146/annurev.genet.40.110405.090613 16771627

[B110] Liu Z. Sekito T. Spirek M. Thornton J. Butow R. A. (2003). Retrograde signaling is regulated by the dynamic interaction between Rtg2p and Mks1p. Mol. Cell 12, 401–411. 10.1016/S1097-2765(03)00285-5 14536080

[B111] Ljungdahl P. O. Daignan-Fornier B. (2012). Regulation of amino acid, nucleotide, and phosphate metabolism in *Saccharomyces cerevisiae*. Genetics 190, 885–929. 10.1534/genetics.111.133306 22419079 PMC3296254

[B112] Lleixà J. Martin V. Portillo Mdel C. Carrau F. Beltran G. Mas A. (2016). Comparison of fermentation and wines produced by inoculation of *Hanseniaspora vineae* and *Saccharomyces cerevisiae*. Front. Microbiol. 7:338. 10.3389/fmicb.2016.00338 27014252 PMC4792884

[B113] Lo W. S. Dranginis A. M. (1996). FLO11, a yeast gene related to the STA genes, encodes a novel cell surface flocculin. J. Bacteriol. 178, 7144–7151. 10.1128/jb.178.24.7144-7151.1996 8955395 PMC178626

[B114] Lorenz M. C. Cutler N. S. Heitman J. (2000). Characterization of alcohol-induced filamentous growth in *Saccharomyces cerevisiae*. Mol. Biol. Cell 11, 183–199. 10.1091/mbc.11.1.183 10637301 PMC14767

[B115] Lorenz M. C. Heitman J. (1998). Regulators of pseudohyphal differentiation in *Saccharomyces cerevisiae* identified through multicopy suppressor analysis in ammonium permease mutant strains. Genetics 150, 1443–1457. 9832522 10.1093/genetics/150.4.1443 PMC1460428

[B116] Madhani H. D. Fink G. R. (1998). The control of filamentous differentiation and virulence in fungi. Trends Cell Biol. 8, 348–353. 10.1016/S0962-8924(98)01298-7 9728395

[B117] Marullo P. Aigle M. Bely M. Masneuf-Pomarède I. Durrens P. Dubourdieu D.. (2007). Single QTL mapping and nucleotide-level resolution of a physiologic trait in wine *Saccharomyces cerevisiae* strains. FEMS Yeast Res. 7, 941–952. 10.1111/j.1567-1364.2007.00252.x 17537182

[B118] McCartney R. R. Schmidt M. C. (2001). Regulation of Snf1 kinase. Activation requires phosphorylation of threonine 210 by an upstream kinase as well as a distinct step mediated by the Snf4 subunit. J. Biol. Chem. 276, 36460–36466. 10.1074/jbc.M104418200 11486005

[B119] Measday V. Moore L. Retnakaran R. Lee J. Donoviel M. Neiman A. M.. (1997). A family of cyclin-like proteins that interact with the Pho85 cyclin-dependent kinase. Mol. Cell. Biol. 17, 1212–1223. 10.1128/MCB.17.3.1212 9032248 PMC231846

[B120] Miller M. B. Bassler B. L. (2001). Quorum sensing in bacteria. Annu. Rev. Microbiol. 55, 165–199. 10.1146/annurev.micro.55.1.165 11544353

[B121] Moffat J. Andrews B. (2004). Late-G1 cyclin-CDK activity is essential for control of cell morphogenesis in budding yeast. Nat. Cell Biol. 6, 59–66. 10.1038/ncb1078 14688790

[B122] Mortimer R. K. Johnston J. R. (1986). Genealogy of principal strains of the yeast genetic stock center. Genetics 113, 35–43. 3519363 10.1093/genetics/113.1.35 PMC1202798

[B123] Mosch H. U. Kubler E. Krappmann S. Fink G. R. Braus G. H. (1999). Crosstalk between the Ras2p-controlled Mitogen- activated Protein Kinase and cAMP Pathways during Invasive Growth of *Saccharomyces cerevisiae*. Mol. Biol. Cell 10, 1325–1335. 10.1091/mbc.10.5.1325 10233147 PMC25273

[B124] Mosch H. U. Roberts R. L. Fink G. R. (1996). Ras2 signals via the Cdc42/Ste20/mitogen-activated protein kinase module to induce filamentous growth in *Saccharomyces cerevisiae*. Proc. Natl. Acad. Sci. U.S.A. 93, 5352–5356. 10.1073/pnas.93.11.5352 8643578 PMC39249

[B125] Niu W. Li Z. Zhan W. Iyer V. R. Marcotte E. M. (2008). Mechanisms of cell cycle control revealed by a systematic and quantitative overexpression screen in *S*. cerevisiae. PLoS Genet 4:e1000120. 10.1371/journal.pgen.1000120 18617996 PMC2438615

[B126] Novo M. Bigey F. Beyne E. Galeote V. Gavory F. Mallet S.. (2009). Eukaryote-to-eukaryote gene transfer events revealed by the genome sequence of the wine yeast *Saccharomyces cerevisiae* EC1118. Proc. Natl. Acad. Sci. U.S.A. 106, 16333–16338. 10.1073/pnas.0904673106 19805302 PMC2740733

[B127] Oppenheim R. D. Creek D. J. Macrae J. I. Modrzynska K. K. Pino P. Limenitakis J.. (2014). BCKDH: the missing link in apicomplexan mitochondrial metabolism is required for full virulence of Toxoplasma gondii and *Plasmodium berghei*. PLoS Pathog. 10:e1004263. 10.1371/journal.ppat.1004263 25032958 PMC4102578

[B128] Orlova M. Ozcetin H. Barrett L. Kuchin S. (2010). Roles of the Snf1-activating kinases during nitrogen limitation and pseudohyphal differentiation in *Saccharomyces cerevisiae*. Eukaryot. Cell 9, 208–214. 10.1128/EC.00216-09 19880754 PMC2805307

[B129] Palecek S. P. Parikh A. S. Kron S. J. (2000). Genetic analysis reveals that FLO11 upregulation and cell polarization independently regulate invasive growth in *Saccharomyces cerevisiae*. Genetics 156, 1005–1023. 11063681 10.1093/genetics/156.3.1005 PMC1461303

[B130] Padilla B. García-Fernández D. González B. Izidoro I. Esteve-Zarzoso B. Beltran G.. (2016). Yeast biodiversity from DOQ priorat uninoculated fermentations. Front. Microbiol. 7:930. 10.3389/fmicb.2016.00930 27379060 PMC4908135

[B131] Palkova Z. Janderova B. Gabriel J. Zikanova B. Pospisek M. Forstová J. (1997). Ammonia mediates communication between yeast colonies. Nature 390, 532–536. 10.1038/37398 9394006

[B132] Pan X. Heitman J. (2002). Protein kinase A operates a molecular switch that governs yeast pseudohyphal differentiation. Mol. Cell. Biol. 22, 3981–3993. 10.1128/MCB.22.12.3981-3993.2002 12024012 PMC133872

[B133] Parsek M. R. Greenberg E. P. (2005). Sociomicrobiology: the connections between quorum sensing and biofilms. Trends Microbiol. 13, 27–33. 10.1016/j.tim.2004.11.007 15639629

[B134] Petrakis T. G. Wittschieben B. O. Svejstrup J. Q. (2004). Molecular architecture, structure-function relationship, and importance of the Elp3 subunit for the RNA binding of holo-elongator. J. Biol. Chem. 279, 32087–32092. 10.1074/jbc.M403361200 15138274

[B135] Polvi E. J. Li X. O'Meara T. R. Leach M. D. Cowen L. E. (2015). Opportunistic yeast pathogens: reservoirs, virulence mechanisms, and therapeutic strategies. Cell. Mol. Life Sci. 72, 2261–2287. 10.1007/s00018-015-1860-z 25700837 PMC11113693

[B136] Prunuske A. J. Waltner J. K. Kuhn P. Gu B. Craig E. A. (2012). Role for the molecular chaperones Zuo1 and Ssz1 in quorum sensing via activation of the transcription factor Pdr1. Proc. Natl. Acad. Sci. U.S.A. 109, 472–477. 10.1073/pnas.1119184109 22203981 PMC3258599

[B137] Querol A. Huerta T. Barrio E. Ramon D. (1992). Dry yeast-strain for use in fermentation of Alicante wines—selection and DNA patterns. J. Food Sci. 57, 183–185. 10.1111/j.1365-2621.1992.tb05451.x

[B138] Ramond E. Gesbert G. Rigard M. Dairou J. Dupuis M. Dubail I.. (2014). Glutamate utilization couples oxidative stress defense and the tricarboxylic acid cycle in *Francisella phagosomal* escape. PLoS Pathog. 10:e1003893. 10.1371/journal.ppat.1003893 24453979 PMC3894225

[B139] Ribéreau-Gayon P. Dubordieu D. Donèche B. Lonvaud A. E. (2000). Handbook of Enology, the Microbiology of Wine and Vinifications. Chichester: John Wiley & Sons.

[B140] Roberts R. L. Fink G. R. (1994). Elements of a single MAP kinase cascade in *Saccharomyces cerevisiae* mediate two developmental programs in the same cell type: mating and invasive growth. Genes Dev. 8, 2974–2985. 10.1101/gad.8.24.2974 8001818

[B141] Robertson L. S. Causton H. C. Young R. A. Fink G. R. (2000). The yeast A kinases differentially regulate iron uptake and respiratory function. Proc. Natl. Acad. Sci. U.S.A. 97, 5984–5988. 10.1073/pnas.100113397 10811893 PMC18545

[B142] Robertson L. S. Fink G. R. (1998a). The three yeast A kinases have specific signaling functions in pseudohyphal growth. Proc. Natl. Acad. Sci. U.S.A. 95, 13783–13787. 10.1073/pnas.95.23.13783 9811878 PMC24897

[B143] Robertson L. S. Fink G. R. (1998b). The three yeast A kinases have specific signaling functions in pseudohyphal growth. Proc. Natl. Acad. Sci. U.S.A. 95, 13783–13787. 10.1073/pnas.95.23.13783 9811878 PMC24897

[B144] Rose M. D. Winston F. Hieter P. (1990). Methods in Yeast Genetics. Cold Spring Harbor, NY: Cold Spring Harbor Laboratory Press.

[B145] Ruiz-Roig C. Noriega N. Duch A. Posas F. de Nadal E. (2012). The Hog1 SAPK controls the Rtg1/Rtg3 transcriptional complex activity by multiple regulatory mechanisms. Mol. Biol. Cell 23, 4286–4296. 10.1091/mbc.E12-04-0289 22956768 PMC3484105

[B146] Rumbaugh K. P. Diggle S. P. Watters C. M. Ross-Gillespie A. Griffin A. S. West S. A.. (2009). Quorum sensing and the social evolution of bacterial virulence. Curr. Biol. 19, 341–345. 10.1016/j.cub.2009.01.050 19230668

[B147] Rupp S. Summers E. Lo H. J. Madhani H. D. Fink G. R. (1999b). MAP kinase and cAMP filamentation signaling pathways converge on the unusually large promoter of the yeast FLO11 gene. EMBO J. 18, 1257–1269. 10.1093/emboj/18.5.1257 10064592 PMC1171216

[B148] Rupp S. Summers E. Lo H. J. Madhani H. Fink G. (1999a). MAP kinase and cAMP filamentation signaling pathways converge on the unusually large promoter of the yeast FLO11 gene. EMBO J. 18, 1257–1269. 10.1093/emboj/18.5.1257 10064592 PMC1171216

[B149] Ryan O. Shapiro R. S. Kurat C. F. Mayhew D. Baryshnikova A. Chin B.. (2012). Global gene deletion analysis exploring yeast filamentous growth. Science 337, 1353–1356. 10.1126/science.1224339 22984072

[B150] Sagot I. Klee S. K. Pellman D. (2002). Yeast formins regulate cell polarity by controlling the assembly of actin cables. Nat. Cell Biol. 4, 42–50. 10.1038/ncb719 11740491

[B151] Saito H. (2010). Regulation of cross-talk in yeast MAPK signaling pathways. Curr. Opin. Microbiol. 13, 677–683. 10.1016/j.mib.2010.09.001 20880736

[B152] Schütz M. Gafner J. (1994). Dynamics of the yeast strain population during spontaneous alcoholic fermentation determined by CHEF gel electrophoresis. J. Appl. Bacteriol. 19, 253–257. 10.1111/j.1472-765X.1994.tb00957.x

[B153] Sekito T. Liu Z. Thornton J. Butow R. A. (2002). RTG-dependent mitochondria-to-nucleus signaling is regulated by MKS1 and is linked to formation of yeast Prion [URE3]. Mol. Biol. Cell 13, 795–804. 10.1091/mbc.01-09-0473 11907262 PMC99599

[B154] Sharma M. Prasad R. (2011). The quorum-sensing molecule farnesol is a modulator of drug efflux mediated by ABC multidrug transporters and synergizes with drugs in *Candida albicans*. Antimicrob. Agents Chemother. 55, 4834–4843. 10.1128/AAC.00344-11 21768514 PMC3186959

[B155] Shemer R. Meimoun A. Holtzman T. Kornitzer D. (2002). Regulation of the transcription factor Gcn4 by Pho85 cyclin Pcl5. Mol. Cell. Biol. 22, 5395–5404. 10.1128/MCB.22.15.5395-5404.2002 12101234 PMC133946

[B156] Sidari R. Caridi A. Howell K. S. (2014). Wild *Saccharomyces cerevisiae* strains display biofilm-like morphology in contact with polyphenols from grapes and wine. Int. J. Food Microbiol. 189, 146–152. 10.1016/j.ijfoodmicro.2014.08.012 25150672

[B157] Sprague G. F. Jr. Winans S. C. (2006). Eukaryotes learn how to count: quorum sensing by yeast. Genes Dev. 20, 1045–1049. 10.1101/gad.1432906 16651650

[B158] Starovoytova A. N. Sorokin M. I. Sokolov S. S. Severin F. F. Knorre D. A. (2013). Mitochondrial signaling in *Saccharomyces cerevisiae* pseudohyphae formation induced by butanol. FEMS Yeast Res. 13, 367–374. 10.1111/1567-1364.12039 23448552

[B159] Suzzi G. Romano P. Zambonelli C. (1984). Flocculation of wine yeasts: frequency, differences, and stability of the character. Can. J. Microbiol. 30, 36–39. 10.1139/m84-006

[B160] Svejstrup J. Q. (2007). Elongator complex: how many roles does it play? Curr. Opin. Cell Biol. 19, 331–336. 10.1016/j.ceb.2007.04.005 17466506

[B161] Toda T. Uno I. Ishikawa T. Powers S. Kataoka T. Broek D.. (1985). In yeast, RAS proteins are controlling elements of adenylate cyclase. Cell 40, 27–36. 10.1016/0092-8674(85)90305-8 2981630

[B162] Tokai M. Kawasaki H. Kikuchi Y. Ouchi K. (2000). Cloning and characterization of the CSF1 gene of *Saccharomyces cerevisiae*, which is required for nutrient uptake at low temperature. J. Bacteriol. 182, 2865–2868. 10.1128/JB.182.10.2865-2868.2000 10781556 PMC101996

[B163] Tu D. Graziano B. R. Park E. Zheng W. Li Y. Goode B. L.. (2012). Structure of the formin-interaction domain of the actin nucleation-promoting factor Bud6. Proc. Natl. Acad. Sci. U.S.A. 109, E3424–3433. 10.1073/pnas.1203035109 23161908 PMC3528572

[B164] Urban J. Soulard A. Huber A. Lippman S. Mukhopadhyay D. Deloche O.. (2007). Sch9 is a major target of TORC1 in *Saccharomyces cerevisiae*. Mol. Cell 26, 663–674. 10.1016/j.molcel.2007.04.020 17560372

[B165] Wang C. García-Fernández D. Mas A. Esteve-Zarzoso B. (2015). Fungal diversity in grape must and wine fermentation assessed by massive sequencing, quantitative PCR and DGGE. Front. Microbiol. 23:1156. 10.3389/fmicb.2015.01156 26557110 PMC4615962

[B166] Wei W. McCusker J. H. Hyman R. W. Jones T. Ning Y. Cao Z.. (2007). Genome sequencing and comparative analysis of *Saccharomyces cerevisiae* strain YJM789. Proc. Natl. Acad. Sci. U.S.A. 104, 12825–12830. 10.1073/pnas.0701291104 17652520 PMC1933262

[B167] Wei Y. Zheng X. F. (2009). Sch9 partially mediates TORC1 signaling to control ribosomal RNA synthesis. Cell Cycle 8, 4085–4090. 10.4161/cc.8.24.10170 19823048 PMC3023923

[B168] Westfall P. J. Ballon D. R. Thorner J. (2004). When the stress of your environment makes you go HOG wild. Science 306, 1511–1512. 10.1126/science.1104879 15567851

[B169] Westman J. O. Franzen C. J. (2015). Current progress in high cell density yeast bioprocesses for bioethanol production. Biotechnol. J. 10, 1185–1195. 10.1002/biot.201400581 26211654

[B170] Williams T. C. Averesch N. J. Winter G. Plan M. R. Vickers C. E. Nielsen L. K.. (2015). Quorum-sensing linked RNA interference for dynamic metabolic pathway control in *Saccharomyces cerevisiae*. Metab. Eng. 29, 124–134. 10.1016/j.ymben.2015.03.008 25792511

[B171] Winkler G. S. Petrakis T. G. Ethelberg S. Tokunga M. Erdjument-Bromage H. Tempst P.. (2001). RNA polymerase II elongator holoenzyme is composed of two discrete subcomplexes. J. Biol. Chem. 276, 32743–32749. 10.1074/jbc.M105303200 11435442

[B172] Woods A. Munday M. R. Scott J. Yang X. Carlson M. Carling D. (1994). Yeast SNF1 is functionally related to mammalian AMP-acitvate protein kinase and regulates acetyl-CoA carboxylase *in vivo*. J. Biol. Chem. 269, 19509–19515. 7913470

[B173] Wuster A. Babu M. M. (2010). Transcriptional control of the quorum sensing response in yeast. Mol. Biosyst. 6, 134–141. 10.1039/B913579K 20024075

[B174] Xu T. Shively C. A. Jin R. Eckwahl M. J. Dobry C. J. Song Q.. (2010). A profile of differentially abundant proteins at the yeast cell periphery during pseudohyphal growth. J. Biol. Chem. 285, 15476–15488. 10.1074/jbc.M110.114926 20228058 PMC2865295

[B175] Young E. T. Pilgrim D. (1985). Isolation and DNA sequence of ADH3, a nuclear gene encoding the mitochondrial isozyme of alcohol dehydrogenase in *Saccharomyces cerevisiae*. Mol. Cell. Biol. 5, 3024–3034. 10.1128/MCB.5.11.3024 2943982 PMC369115

[B176] Zaman S. Lippman S. I. Zhao X. Broach J. R. (2008). How Saccharomyces Responds to Nutrients. Annu. Rev. Genet. 42, 27–81. 10.1146/annurev.genet.41.110306.130206 18303986

[B177] Zupan J. Raspor P. (2008). Quantitative agar-invasion assay. J. Microbiol. Methods 73, 100–104. 10.1016/j.mimet.2008.02.009 18358550

