# Supplementary material for: Role of Mitochondrial Retrograde Pathway in Regulating Ethanol-Inducible Filamentous Growth in Yeast
Source: Front Physiol. 2017 Mar 29;8:148. doi: 10.3389/fphys.2017.00148 (PMC5372830; doi:10.3389/fphys.2017.00148)
Supplement: Supplementary file 1 [file Presentation1.PDF]

SUPPLEMENTAL FIGURE LEGENDS

Figure S1. Pseudohyphal growth and *FLO11* expression in conditions with different nitrogen concentrations. A) Pseudohyphal growth of micro-colonies. Cells were grown for 3 days in minimal medium (MM) at 30˚C, diluted by a factor of 10^6 and spotted onto SAD, SLAD, and SLAG media. Plates were incubated for 5 days. Colonies were examined by microscopy at 5X magnification. Representative images are shown. Bars, 200 microns. **B)** The expression of *FLO11* was examined by qPCR in four strains Σ1278b, QA23, T73 and P5. Cells were grown in SAD (grey bars) and in SLAD (orange bars) for 2h. Error bars represent the standard error between samples. Asterisk denotes a p-value of <0.01.

Figure S2. Characterization of ethanol concentration on filamentous growth and growth rate. A) The effect of ethanol on invasive growth of cells grown on different media in the QA23 strain. **B)** Ethanol concentration and invasive growth in SLAD media for the indicated yeast strains. **C)** Impact of different concentrations of ethanol on growth of wild-type *MAT***a** / *MAT*α Σ1278b cells (PC344) over a time-course experiment.

Figure S3. Impact of the fusel alcohol tryptophol on filamentous growth of wine yeast. Equal concentrations of cells (OD_{600nm} 2) were spotted in 10µL aliquots onto SAD and SLAD media with or without 500µM tryptophol (TRP-OH). Plates were incubated for 5 days at 30ºC and washed in a stream of water. Colonies before washing grew to approximately equal levels and were similar in appearance (not shown).

Figure S4. Invasive growth phenotypes of signaling mutants. A) Cells were spotted onto YPD media. Plates were incubated for 2 days at 30ºC and washed in a stream of water. **(B)** Plate-washing assay of colonies grown in SLAD medium or SLAD medium supplemented with ethanol (2% v/v) or hydroxyurea (100 mM). **(C)** Cells lacking Rtg2p grown under the same conditions as panel B. Bar, 20 microns.

Figure S5. Phenotypes of a collection of mutants involved in metabolism were tested for ethanol-dependent filamentous growth. (A) Wild-type cells and the indicated mutants were spotted onto nitrogen-limited medium (SLAD) with or without 2% ethanol (v/v). Plates were photographed, washed in stream of water, and photographed again. Bar, 5 millimeters. **(B)** Quantitation of invasive growth in panel A by densitometry. The average values of three independent replicates are shown. Error bar represents standard difference between samples. Single asterisk denotes the p-value of <0.01 compared to the no ethanol control (blue to orange bars). Double asterisk denotes p-value of <0.01 compared to wild-type ethanol control. **(C)** Colony peripheries from the plates in panel A were photographed at 20X magnification. Bar, 25 microns. Arrows mark examples of pseudohyphae.

Fig._S1

A

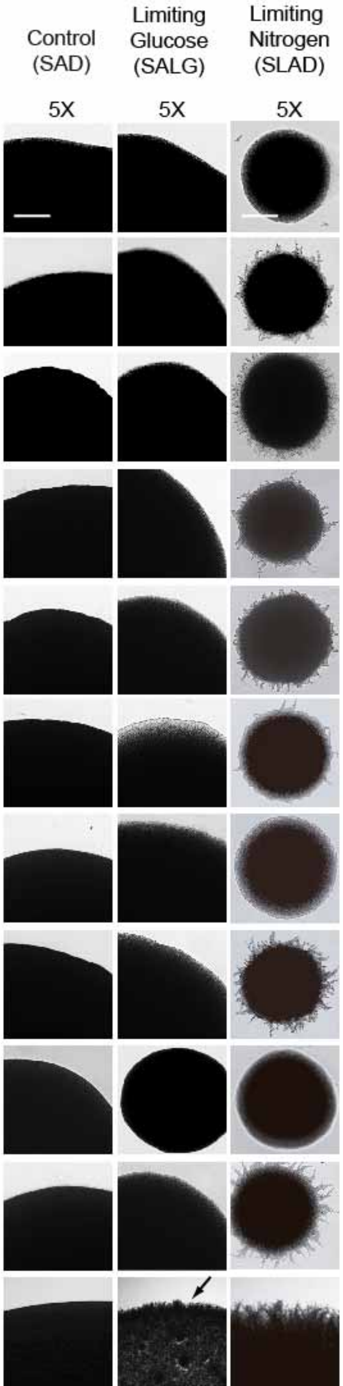
Control (SAD)
Limiting Glucose (SALG)
Limiting Nitrogen (SLAD)
5X
5X
5X

B

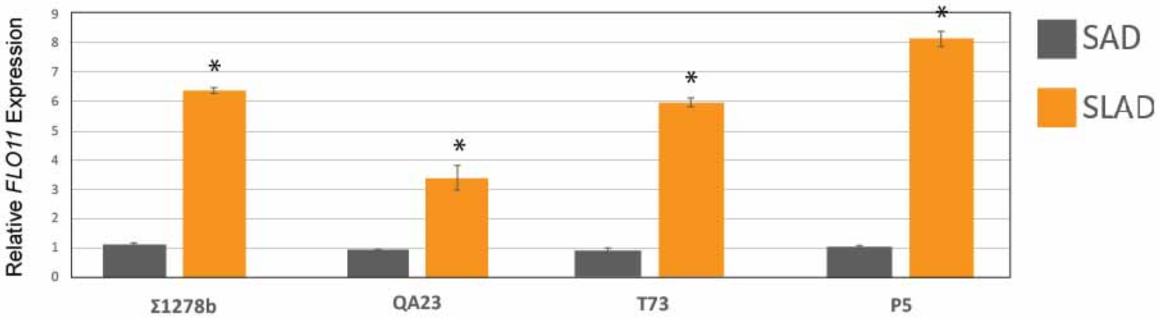
Relative FLO11 Expression
SAD
SLAD
Σ1278b
QA23
T73
P5
*
0
1
2
3
4
5
6
7
8
9

Fig._S2

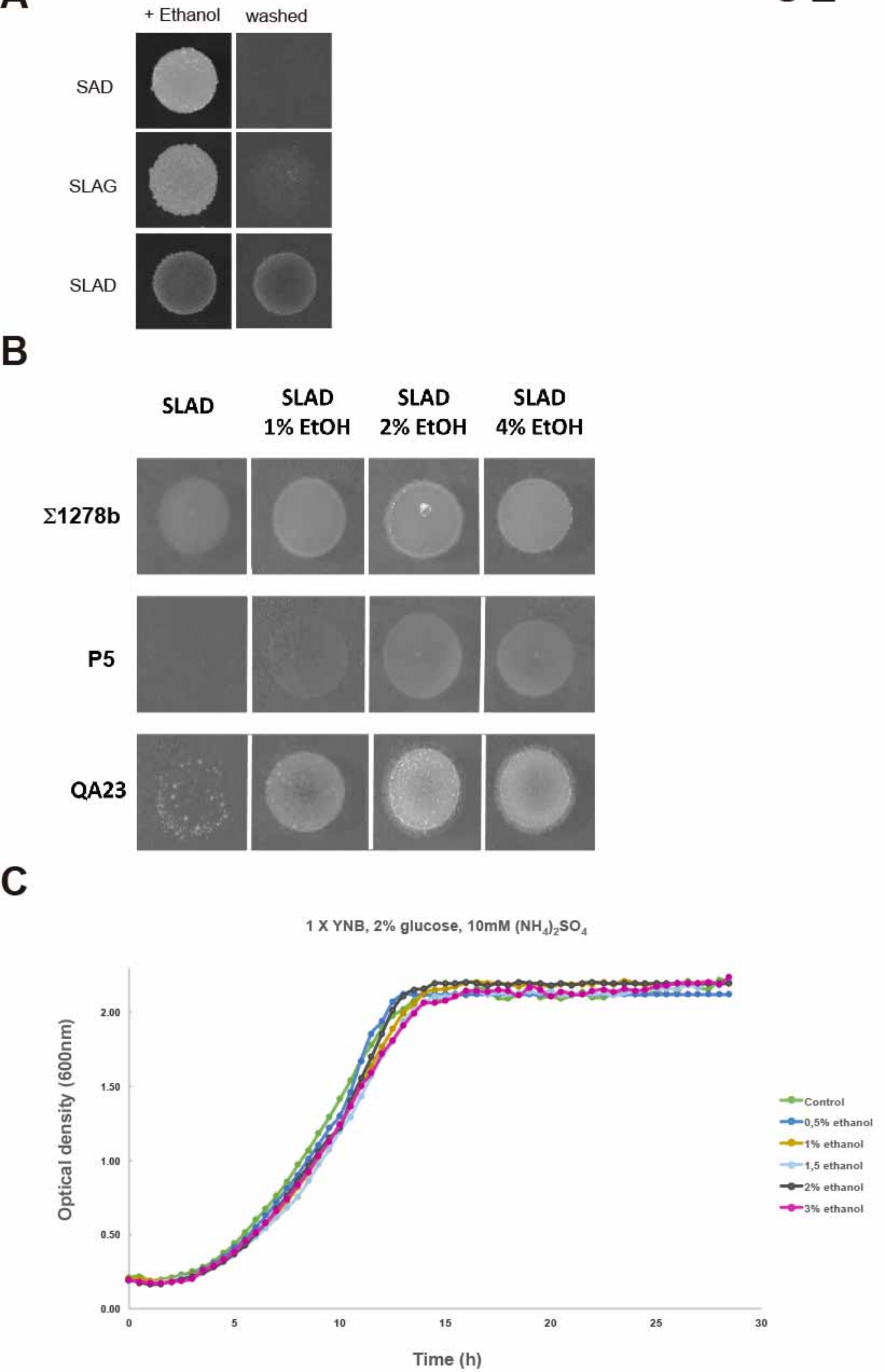
A
+ Ethanol
washed
SAD
SLAG
SLAD
B
SLAD
SLAD 1% EtOH
SLAD 2% EtOH
SLAD 4% EtOH
Σ1278b
P5
QA23
C
1 X YNB, 2% glucose, 10mM $(NH_4)_2SO_4$
Optical density (600nm)
0.00
0.50
1.00
1.50
2.00
0
5
10
15
20
25
30
Time (h)
Control
0,5% ethanol
1% ethanol
1,5 ethanol
2% ethanol
3% ethanol

Fig._S3

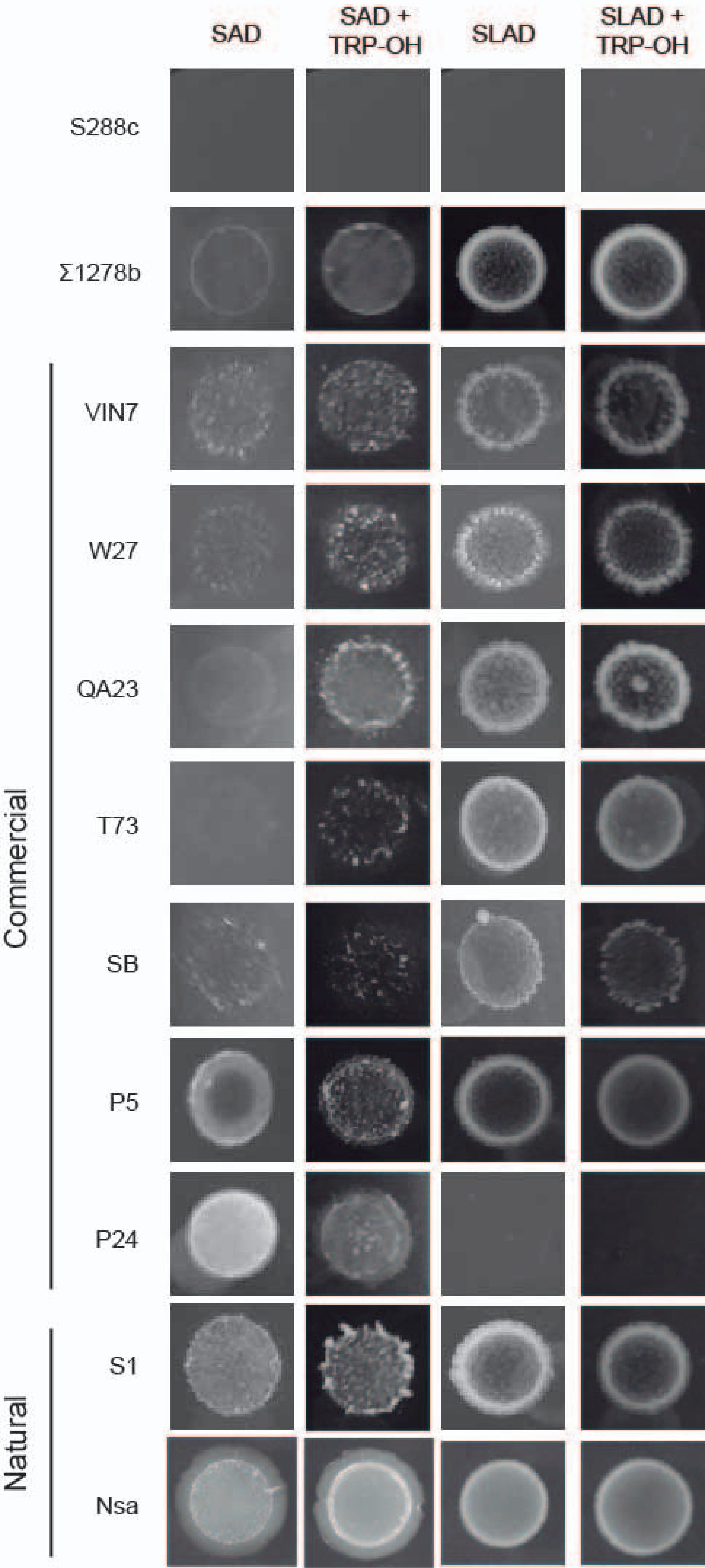

SAD
SAD + TRP-OH
SLAD
SLAD + TRP-OH
S288c
Σ1278b
VIN7
W27
QA23
T73
SB
P5
P24
S1
Nsa
Commercial
Natural

Fig._S4

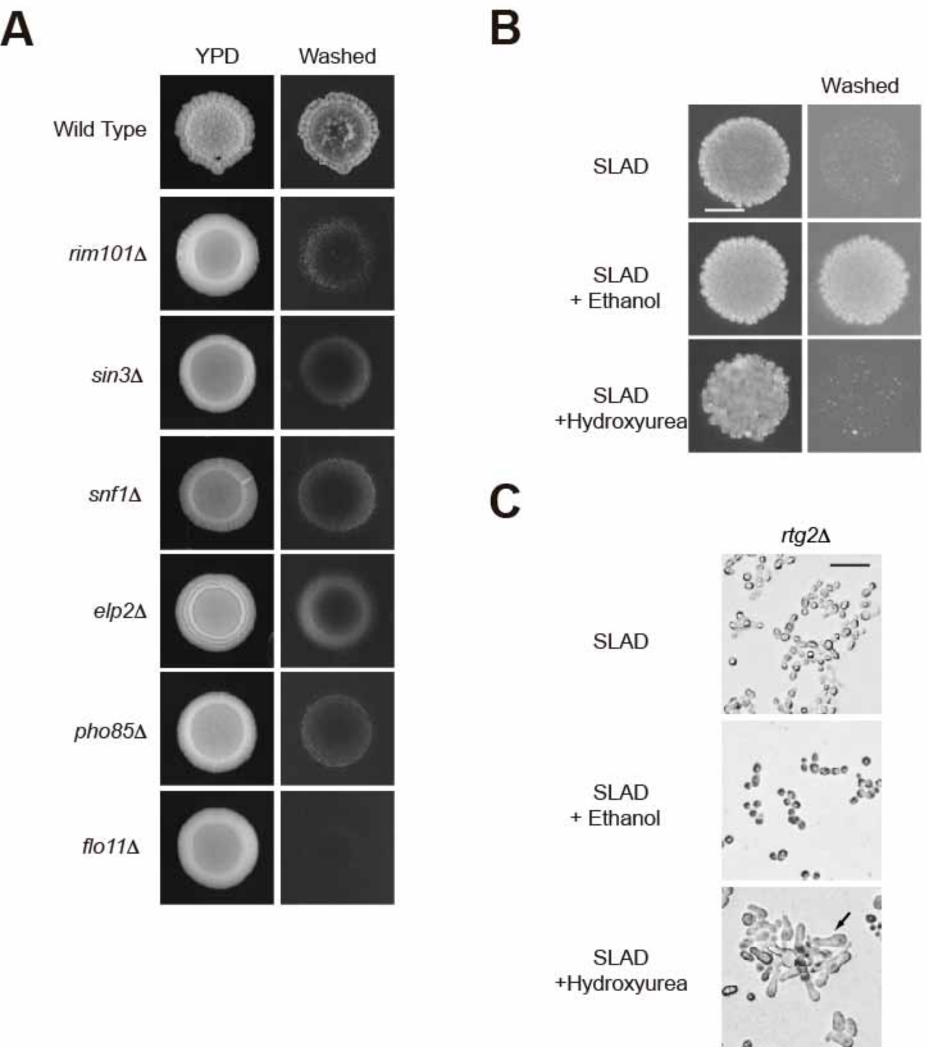
A
YPD
Washed
Wild Type
rim101Δ
sin3Δ
snf1Δ
elp2Δ
pho85Δ
flo11Δ
B
Washed
SLAD
SLAD
+ Ethanol
SLAD
+Hydroxyurea
C
rtg2Δ
SLAD
SLAD
+ Ethanol
SLAD
+Hydroxyurea

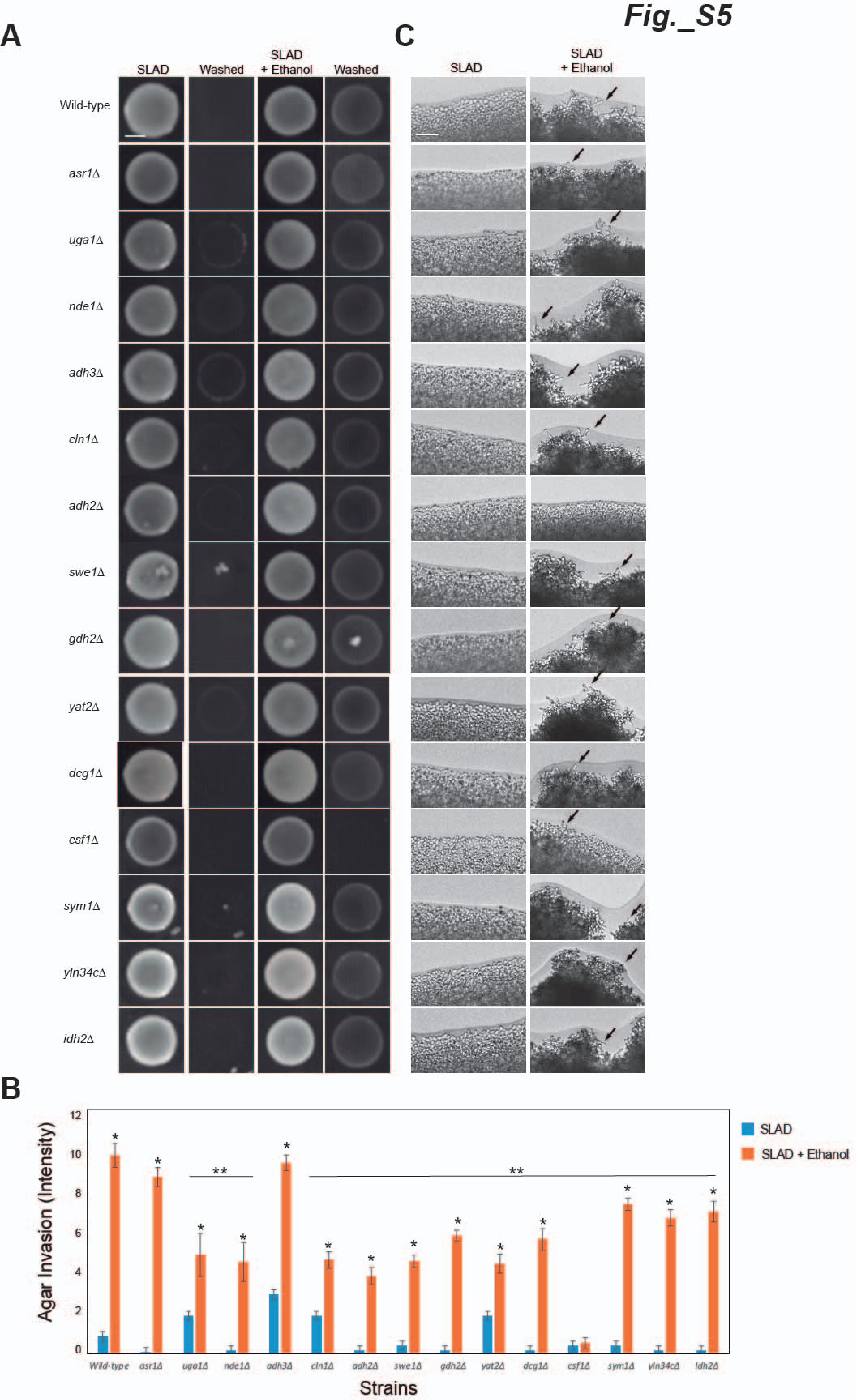
Fig._S5
A
SLAD
Washed
SLAD + Ethanol
Washed
Wild-type
asr1Δ
uga1Δ
nde1Δ
adh3Δ
cln1Δ
adh2Δ
swe1Δ
gdh2Δ
yat2Δ
dcg1Δ
csf1Δ
sym1Δ
yln34cΔ
idh2Δ
C
SLAD
SLAD + Ethanol
B
Agar Invasion (Intensity)
Strains
SLAD
SLAD + Ethanol
Wild-type
asr1Δ
uga1Δ
nde1Δ
adh3Δ
cln1Δ
adh2Δ
swe1Δ
gdh2Δ
yat2Δ
dcg1Δ
csf1Δ
sym1Δ
yln34cΔ
idh2Δ
